# High-throughput LacZ/CPRG assay for identifying Gram-negative envelope-targeting antibiotics

**DOI:** 10.3389/fmicb.2026.1779546

**Published:** 2026-09-23

**Authors:** Fardin Ghobakhlou, Larbi Mokhtari, Tom A. Pfeifer, Catherine Paradis-Bleau

**Affiliations:** 1 Department of Microbiology, Infectiology and Immunology, Faculty of Medicine, Université de Montréal, Montreal, QC, Canada; 2 Biofactorial, Life Sciences Center, University of British Columbia, Vancouver, BC, Canada

**Keywords:** antibiotic resistance, bacterial envelope integrity, combination therapy, confocal microscopy, drug discovery, Gram-negative bacteria, high-throughput screening (HTS), LacZ/CPRG assay

## Abstract

**Background:**

**Objectives:**

We aimed to develop a liquid-based high-throughput screening (HTS) assay to identify small molecules that target GN bacterial envelope integrity and to assess their antibacterial potential, distinct from traditional viability-based screens.

**Methods:**

Envelope disruption in *Escherichia coli* and *Pseudomonas aeruginosa* was assessed using a β-galactosidase (LacZ)/CPRG reporter assay in LB at 37 °C. The assay was validated by screening the LOPAC^1280^ and KD_2_^4761^ compound libraries. Hit compounds were further characterized by concentration-response analysis, determination of permeabilization constants (K_50_), chloramphenicol combination permeabilization assays, minimal inhibitory concentration (MIC) measurements, and microscopy at sub-MICs. Confocal microscopy served as an orthogonal validation method.

**Results:**

The assay demonstrated robust, reproducible performance across independent optimization runs, as evidenced by high Z’-factor and signal-to-noise (S/N) ratios. Screening identified 72 active compounds, including β-lactams and four non-antibiotic molecules (suloctidil, isorotenone, alexidine, and thiram) that showed concentration-dependent increases in CPRG reporter signal, consistent with envelope permeabilization. Thiram was excluded due to its fungicidal use, and the remaining three compounds were further characterized. Alexidine showed the most potent activity, with the lowest K_50_ (2.7 × 10^−3^ mM) and MICs of 0.004 mM for *E. coli* and 0.015 mM for *P. aeruginosa*. Suloctidil and isorotenone induced spherical cell morphology, whereas alexidine induced a filamentous phenotype, indicative of envelope disruption. The assay also identified compounds with both antimicrobial activity and potential antibiotic adjuvant effects. Apicillin, alexidine, and suloctidil enhanced chloramphenicol activity against *E. coli* MG1655, consistent with increased envelope permeability.

**Conclusion:**

The LacZ/CPRG reporter assay enables rapid identification of compounds targeting bacterial envelopes, including novel molecules active against GN pathogens, providing a high-throughput functional platform that complements established permeability assays and accelerates discovery of new therapeutics.

## 1 Introduction

Infectious diseases cause 1.5 billion cases and 4.6 million deaths each year (Taylor et al., 2001; Lindahl and Grace, 2015; Tang et al., 2023). Multidrug-resistant organisms (MDROs), especially Gram-negative bacteria (GN), worsen outcomes by hindering treatment and increasing morbidity and mortality (Brown and Wright, 2016). MDROs caused 700,000 deaths in 2014, with projections reaching 10 million by 2050 (O'neill, 2014). They lead to 160,000 deaths annually in the U.S. and 138,000 in Europe (Laxminarayan et al., 2016; Paitan, 2018; Burnham et al., 2019), and healthcare costs exceed $35 billion (Fulton et al., 2023). The global burden may approach $100 trillion by mid-century (Burnham et al., 2019), with U.S. costs at $7.2–14.9 billion in 2016 (Forrester et al., 2022). GN cause ~50% of bacterial infections and have higher mortality rates than Gram-positive (GP) organisms (Fulton et al., 2023). Treating multidrug-resistant GN is challenging because their outer membrane blocks antibiotics (Delcour, 2009). Antimicrobial resistance (AMR) arises from intrinsic or acquired mechanisms, including inactivating enzymes and mutations (Alekshun and Levy, 2007; Ruppé et al., 2015). The spread of MDROs, such as carbapenem-resistant *Enterobacteriaceae*, further limits treatment options (Nordmann et al., 2011; Brown and Wright, 2016; Paradis-Bleau et al., 2014). Advances in GN envelope biogenesis (Ruiz et al., 2006; Paradis-Bleau et al., 2014) underscore the need for improved antibiotic susceptibility tests (AST), targeted drugs, and effective diagnostics (Brown and Wright, 2016). Current AST methods, including conventional and high-throughput screening (HTS), remain time-consuming (Hattab et al., 2024). New approaches—electrochemical-based PCR-less (Chen et al., 2021), photonic (Meulebroeck et al., 2024), imaging (Scherer et al., 2021; Song and Lei, 2021), and Raman scattering microscopy (Han et al., 2023)—along with molecular tools such as whole-genome sequencing (van Belkum et al., 2019), microfluidics (Scherer et al., 2021; Lehnert and Gijs, 2024), proteomics (Blumenscheit et al., 2021), and machine learning-assisted AST (Gajic et al., 2022; Sturm et al., 2024) are improving resistance detection. Emerging technologies, including HTS and whole-cell assays (Sarker et al., 2007; Brackett et al., 2014; McLeod et al., 2015; Paytubi et al., 2017; Syal et al., 2017; Kulesa et al., 2018), have been developed to identify compounds that inhibit bacterial growth and cell wall biosynthesis (Ang et al., 2015; Nayar et al., 2015; Raneri et al., 2015; Pulido et al., 2016; Avesar et al., 2017; Collia et al., 2018; Haynes et al., 2018; Naclerio and Sintim, 2020). Despite progress in diagnostics (Miller et al., 2019; World Health Organization, 2019; Park et al., 2019; Pitruzzello et al., 2019), universal platforms face limits due to cost and the challenge of targeting GN outer membranes (van Belkum et al., 2019). With rising AMR and 50% of antibiotics prescribed without confirmed pathogen identification (Vasala et al., 2020), rapid high-throughput methods are urgently needed to find new small molecules that target GN envelopes. This underscores the importance of functional screening approaches that can efficiently evaluate envelope integrity in a high-throughput manner.

We present a novel biochemical HTS assay to detect small molecules that disrupt GN envelope integrity, distinct from traditional viability-based screens. The LacZ/CPRG [β-galactosidase (LacZ)/chlorophenol red-β-D-galactopyranoside (CPRG)] assay, optimized in liquid Luria-Bertani (LB) culture, monitors envelope permeabilization in *E. coli* MG1655, a representative GN model (Paradis-Bleau et al., 2014). CPRG, normally excluded by an intact envelope, enters the cytoplasm upon membrane disruption and is hydrolyzed by β-galactosidase, the enzyme encoded by the *E. coli lacZ* gene, producing a quantitative chromogenic signal at 575 nm. β-Galactosidase activity serves as a proxy for envelope permeability via CPRG hydrolysis. Although it does not distinguish between outer- and inner membrane perturbations, the assay provides a rapid functional assessment of overall envelope integrity. To complement this population-level readout, we also developed a DAPI/FITC/CTC triple-staining confocal microscopy approach as a proof of concept for distinguishing membrane permeabilization from bacterial viability at single-cell resolution.

The assay complements existing methods for assessing bacterial membrane permeability and viability. Accordingly, it is intended to complement established assays, including the NPN uptake assay for GN outer membrane permeability (Helander and Mattila-Sandholm, 2000), propidium iodide (PI)/SYTOX staining for cellular membrane integrity (Mortimer et al., 2000), and the Nitrocefin chromogenic cephalosporin assay, which independently quantifies β-lactamase activity across diverse bacterial genera (O'Callaghan et al., 1972). The LacZ/CPRG assay detects a color change upon envelope disruption, enabling high-throughput screening of compound libraries. We validated the assay using pathogenic strains, including *E. coli* and *Pseudomonas aeruginosa*, and applied it to screen the LOPAC^1280^ and KD_2_ (4,761 compounds) libraries (Ruiz et al., 2006; Sarker et al., 2007; Paradis-Bleau et al., 2014). The screen identified active compounds, including β-lactams, suloctidil, isorotenone, and alexidine, that alter the CPRG signal in a manner consistent with envelope permeabilization, revealing molecules with antibacterial activity and potential antibiotic adjuvant properties.

This approach enables identification of small molecules that induce envelope permeabilization either through direct antibacterial activity or by enhancing antibiotic entry. The LacZ/CPRG platform is rapid, sensitive, quantitative, and scalable for high-throughput screening. To our knowledge, this is the first application of a LacZ/CPRG-based screening strategy to identify antibacterial compounds that target GN bacterial envelopes.

## 2 Materials and methods

### 2.1 Bacterial strains and growth conditions

*E. coli* MG1655 was used as the wild-type (WT) control, and the envelope-defective mutant MG1655 (rfb-50, O-antigen defective) (Raetz and Whitfield, 2002) was used for HTS optimization and is referred to as *Δ*rfb-50 in figures for brevity. Validation was performed with Enterohemorrhagic (EHEC) *E. coli* (EDL933, O157:H7), Enteropathogenic (EPEC) *E. coli* (E2348/69, O127:H6Str) (Thomassin et al., 2012), Uropathogenic (UPEC) *E. coli* (CFT073, O6:K2:H1) (Sarkar et al., 2014), and *P. aeruginosa* PAO1. Strains were cultured in LB medium at 37 °C.

### 2.2 LacZ/CPRG reporter assay optimization for 96-well and 384-well plates

The LacZ/CPRG assay, originally developed by Paradis-Bleau et al. (2014), was optimized in liquid media and adapted to 96-well and 384-well plates to facilitate high-throughput screening of small molecules targeting GN bacterial envelope integrity (Figure 1A). Ampicillin (positive control), chloramphenicol (orthogonal negative control), and LB + *E. coli* (negative/baseline control) were used for assay optimization (Spratt, 1975; Sicard et al., 2014). Unless otherwise specified, compounds and antibiotics were prepared in DMSO, and negative/baseline controls received an equivalent volume of DMSO. Ampicillin, chloramphenicol, CPRG, and isopropyl β-D-thiogalactopyranoside (IPTG) were titrated in two independent experiments to determine optimal working concentrations. Fresh LB and LB containing *E. coli* MG1655 were included as contamination and background negative controls, respectively. A subset of control experiments including LB + cells + CPRG confirmed that spontaneous CPRG hydrolysis was minimal under assay conditions.

**Figure 1:**
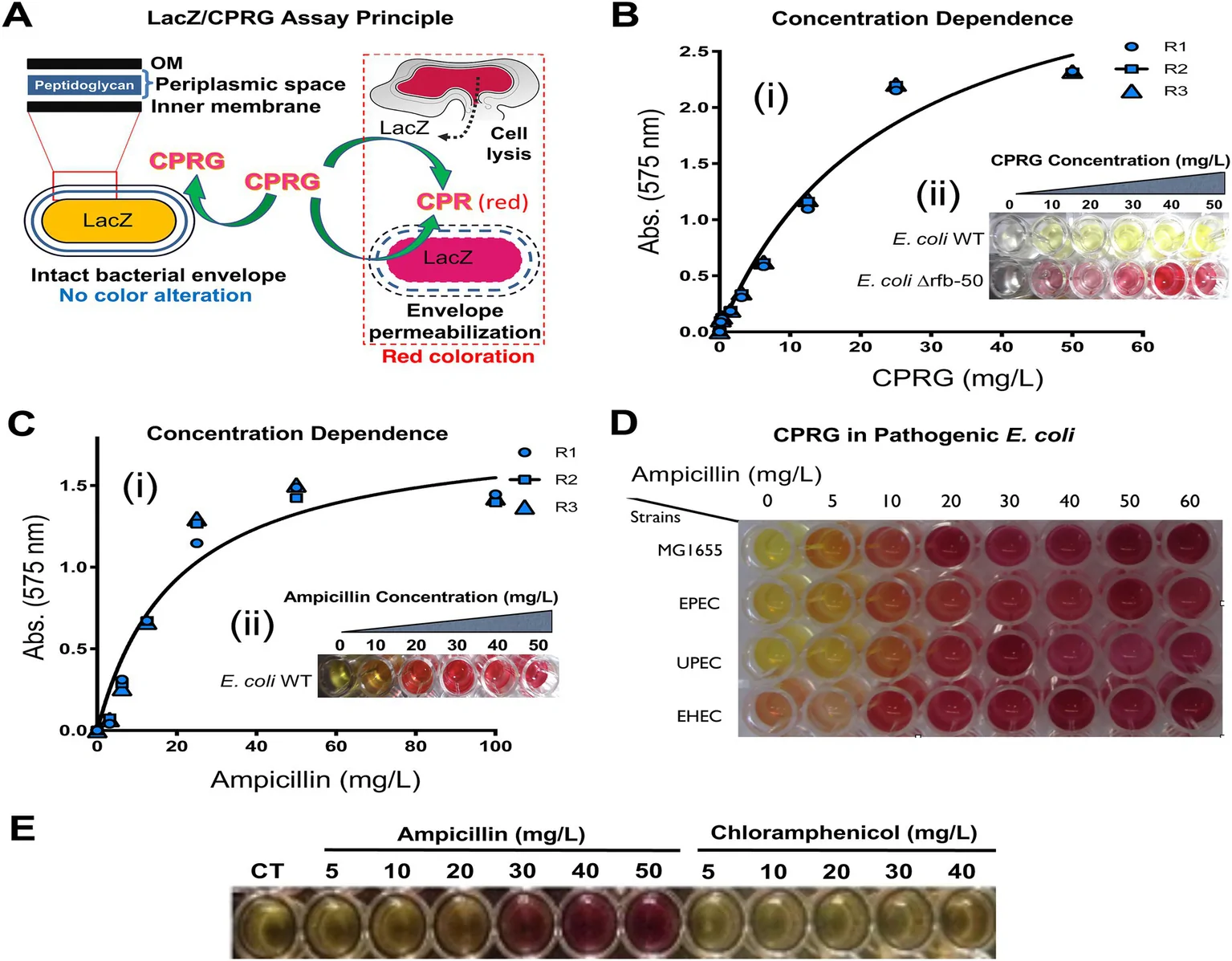
Optimization and validation of a LacZ/CPRG envelope integrity assay in liquid medium. **(A)** Schematic of the LacZ/CPRG reporter assay. CPRG is excluded by an intact Gram-negative envelope; upon envelope disruption, it enters the cytoplasm and is hydrolyzed by β-galactosidase (LacZ) to chlorophenol red, generating a chromogenic signal measurable at OD_575_. **(B)** CPRG optimization in 96-well format (100 μL). (i) Nonlinear regression analysis showing concentration-dependent CPRG signal, with an apparent half-maximal response concentration of ~25 mg/L. (ii) Representative microplate wells showing assay specificity and optimal CPRG concentration using WT and envelope-defective *E. coli* (∆rfb-50). Signal remained stable for >3 h, and 40 mg/L CPRG was selected for subsequent screening. **(C)** Ampicillin optimization under identical conditions. (i) Nonlinear regression analysis showing concentration-dependent response to ampicillin, with an apparent half-maximal response concentration of ~21 mg/L. (ii) Representative microplate wells showing robust signal generation with 40 mg/L ampicillin and 40 mg/L CPRG. **(D)** Evaluation in pathogenic *E. coli* strains with intact LPS showing ampicillin-dependent CPRG response, with highest sensitivity in EHEC and lowest in UPEC relative to the O-antigen-defective MG1655 (*∆*rfb-50) control. **(E)** Representative CPRG assay wells showing strong color development with ampicillin at 40 mg/L CPRG, whereas chloramphenicol, used as an orthogonal negative control, produced no detectable signal.

CPRG absorbance detection consistency was evaluated by comparing Synergy and Varioskan plate readers using serial dilution reactions in 200 μL volumes (Supplementary Figure S1). Signal discrimination at the CPRG absorbance readout (575 nm) was assessed to evaluate assay resolution and minimize background interference for envelope-targeting activity. To determine whether bacterial concentration could be reduced in the screening assay, several OD_600_ and OD_660_ values (0.5, 0.4, 0.3, 0.2, 0.1, and 0.05) were tested. Several CPRG concentrations (80, 60, 40, 20, 10, and 5 mg/L) were also tested in parallel. The OD could be reduced to OD_660_ = 0.05, though at OD_600_ = 0.05, a longer incubation time (5 h versus 3 h) was needed to achieve maximum signal. A final CPRG concentration of 40 mg/L was optimal for the assay. Overnight *E. coli* cultures were grown in fresh LB to mid-log phase (OD_600_ ≈ 0.5), supplemented with CPRG, and tested in 200 μL and 100 μL reaction volumes using ampicillin as a positive control and chloramphenicol as an orthogonal negative control. LB + *E. coli* served as a negative/baseline control, and LB alone was included to monitor contamination. Absorbance at 575 nm was used to compare signal discrimination across conditions, confirming clear separation between active and control groups (Supplementary Figure S2).

To optimize assay miniaturization, reaction volumes were reduced from 200 μL to 100 μL and 50 μL. Absorbance kinetics at 575 nm were monitored for up to 150 min in both 96- and 384-well formats (Supplementary Figures S3, S4), and signal reached a plateau under all conditions. Additional measurements at 3 h and overnight were performed to assess long-term signal stability, using the same control set (ampicillin, LB + *E. coli*, chloramphenicol, and LB alone). Concentration–response curves were fitted by nonlinear regression using a Michaelis–Menten–type model to estimate apparent half-maximal response concentrations. Assay performance metrics (Z′-factor, S/N, and S/B) were determined from positive and baseline control distributions across independent assay preparations and technical replicates.

#### 2.2.1 96-well plate format optimization

For 96-well plates (Nunc #167314), a fresh *E. coli* MG1655 colony was grown overnight in 4 mL LB + 100 μM IPTG at 37 °C (250 rpm), then diluted 1:50 into 25 mL LB + 100 μM IPTG, incubated to mid-log (OD_600_ = 0.5, ~90 min), and supplemented with 40 mg/L (40 μg/mL) CPRG. Cultures (200 μL) were transferred to wells. LB plus bacterial cells served as baseline controls. To evaluate assay performance, ampicillin (40 mg/L; positive control) and chloramphenicol (40 mg/L; orthogonal control) were included. Plates were shaken at room temperature, color development was measured at OD_575_ using a microplate reader (PowerWave XS, Biotek) and performance was assessed via Z’, Z-factor, and S/N ratios (Zhang et al., 1999).

#### 2.2.2 384-well plate preparation and compound transfer for HTS

For high-throughput screening (HTS), the assay was miniaturized to 384-well plates (Thermo Fisher Scientific #3701). The protocol was identical to that for the 96-well format unless otherwise noted. An overnight *E. coli* MG1655 culture was diluted 1:50 (2 mL into 100 mL LB + 100 μM IPTG in a 2 L flask) and grown to OD_600_ = 0.6 (~2 h). Two 384-well assay plates were prepared. For the first 384-well plate, one half of the plate was preloaded with 25 μL/well of LB containing ampicillin as the positive control. Ampicillin was prepared as a 30 mg/mL stock solution in DMSO and dispensed at 70 nL per well. The second half of the plate was preloaded with 25 μL/well of LB containing bacterial cells as the negative/ untreated baseline control. The final column contained LB with DMSO as a vehicle/contamination control. For the second 384-well plate, ampicillin and chloramphenicol served as positive and orthogonal negative controls, respectively. One half of the plate received ampicillin, and the other half received chloramphenicol. Both compounds were prepared as 30 mg/mL stock solutions in DMSO and dispensed at 70 nL per well. Vehicle-control wells received an equivalent volume of DMSO to maintain the final DMSO concentration at 0.14% (v/v).

A separate 30 mL bacterial culture was prepared and supplemented with CPRG at 80 mg/L. Using a Thermo Combi liquid handler, 25 μL of the CPRG-containing bacterial culture was dispensed into each well containing preloaded compounds or controls. The final assay volume was 50 μL, yielding final concentrations of 40 mg/L CPRG and ~40 mg/L ampicillin or chloramphenicol per well (Supplementary Figure S5). For assay optimization, a parallel condition with 20 mg/L CPRG was also evaluated at both 3 h and overnight. Plates were incubated at room temperature with agitation, and OD_575_ was measured at 3 h and after overnight incubation to detect both rapid and delayed cell envelope permeabilization. Assay performance was evaluated using Z’-factor, S/B, and S/N derived from baseline and positive control distributions. Pathogenic *E. coli* strains (EHEC EDL933, O157:H7; EPEC E2348/69, O127:H6; UPEC CFT073, O6: K2:H1) (Thomassin et al., 2012; Sarkar et al., 2014) were tested and compared with MG1655 (rfb-50, O-antigen defective strain) (Raetz and Whitfield, 2002) under identical conditions.

### 2.3 Automation of the CPRG assay by screening the LOPAC^1280^ library

The LOPAC^1280^ library (Sigma; 4 plates) of well-characterized compounds was used to evaluate the specificity and sensitivity of the automated CPRG assay in a 384-well format. Compounds were pin transferred using a PlateMate Plus with an FP3 pin tool (final concentration: 6.0 μM/well). Bacterial cultures (30 mL) were prepared in LB supplemented with CPRG (80 mg/L), with a separate ampicillin-supplemented culture (40 mg/L) prepared specifically for the positive control. For plate setup, 25 μL of the ampicillin- and CPRG-supplemented culture was dispensed into all other wells, including column 24 (cell-free background blank, LB + CPRG, no bacteria) and the compound-test wells (final CPRG concentration: 40 mg/L per well). Plates were incubated at room temperature on a shaker, and OD_575_ was measured after 3 h and overnight using a PowerWave XS reader (BioTek). Wells containing LB + *E. coli* + CPRG (negative/baseline control) were defined as 100% signal, while column 24 (LB + CPRG, no bacteria) served as the cell-free background blank for background subtraction. Normalized CPRG response was calculated as: % CPRG signal = [(sample − blank)/(negative control − blank)] × 100. As a preliminary pilot screen, the LOPAC^1280^ assay was performed in a single-point, high-throughput format to assess assay performance, dynamic range, and reproducibility prior to large-scale KD_2_ library screening.

### 2.4 Automation parameters of the CPRG assay in HTS using the KD_2_ library

The KD_2_ library (4,761 compounds, 16 plates) comprises four sub-libraries: the Prestwick library (1,120 compounds, 4 plates), the MicroSource library (2,000 compounds, 7 plates), the Sigma-LOPAC library (1,280 compounds, 4 plates), and the BIOMOL library (361 compounds, 1 plate). Note that the Sigma-LOPAC sub-library within KD_2_ is the same library screened independently in Section 2.3. The KD_2_ library was screened using the same procedure as the LOPAC^1280^ library. All assay wells contained LB medium, bacterial cells, and CPRG substrate. LB + CPRG wells (no bacteria) served as the cell-free background blanks for background subtraction. Wells containing LB + bacteria + CPRG (negative/baseline control) were defined as 100% signal. Normalized CPRG response was calculated as: % CPRG signal = [(sample − blank)/(negative control − blank)] × 100. Assay performance was evaluated at 20 and 40 mg/L CPRG after 3 h and overnight incubation to identify optimal conditions for HTS, with 40 mg/L selected for screening. Quality-control metrics, including Z′-factor, signal-to-noise ratio, and signal-to-background ratio, were calculated from control wells across all plates (Table 1).

**Table 1:** Statistical performance parameters for 20 and 40 mg/L CPRG after 3-h and overnight incubation.

Incubation	CPRG 20 mg/L	CPRG 40 mg/L
3 h	Z’-factor = 0.8	Z’-factor = 0.9
	S/B = 2.3	S/B = 4.8
	S/N = 81	S/N = 194
Overnight	Z’-factor = 0.1	Z’-factor = 0.6
	S/B = 1.4	S/B = 1.8
	S/N = 5.9	S/N = 9.4

$$\begin{array}{l} Z^{\prime }-\text{factor}=1-\frac{3\mathrm{SD}\;\text{of sample}+3\mathrm{SD}\;\text{of control}}{|\text{mean of sample}-\text{mean of control}|}\;\\ S/B=\frac{\text{mean signal}}{\text{mean background}}\;\\ S/N=\frac{\text{mean signal}-\text{mean background}}{\text{standard deviation of background}}. \end{array}$$

### 2.5 Cherry-picking for confirmation of 72 active envelope-targeting compounds

Compounds identified in the LOPAC and KD_2_ screens were cherry-picked for validation. Compounds (0.5 μL; 45 μM final concentration) were transferred to assay plates. Absorbance at OD_575_ and OD_660_nm was measured every 30 min for 3 h, then hourly for 11 h to concurrently assess chromogenic product formation and culture turbidity. This dual-wavelength approach enabled normalization to cell density and reduced optical background interference. Background absorbance was subtracted, and CPRG signals were analyzed as percent signal relative to LB + bacterial cells (no compound). Hit compounds identified from primary screening were subsequently evaluated in independent biological replicates (*n* > 3) to confirm reproducibility.

### 2.6 Concentration-response curves

To assess whether assay signal depends on bacterial cell density or incubation time, a pilot experiment tested four primary hit compounds (isorotenone, alexidine, suloctidil, thiram) at a fixed concentration of [0.025 mM (25 μM)], at two initial bacterial densities (OD_600_ = 0.1 and 0.4) and two timepoints (3 h and 5 h). Based on pilot results showing no dependence on cell density or timepoint, the main concentration-response study titrated six compounds (vancomycin, cefaclor, cefsulodin, isorotenone, suloctidil, alexidine) from [2.5 mM (2,500 μM)] to [0.0025 mM (2.5 μM)] at a fixed density of OD_600_ = 0.1. Compounds were prepared as [20–25 mM (20,000–25,000 μM)] stocks in DMSO; DMSO served as vehicle control.

For all experiments, overnight *E. coli* cultures were diluted 1:50 into sterile LB supplemented with [0.1 mM (100 μM)] IPTG and incubated at 37 °C, 250 rpm until reaching a starter density of OD_600_ = 1 (~2–3 h). This starter culture was then diluted to the appropriate assay working density (OD_600_ = 0.1 or 0.4) and combined with compound in 384-well assay plates (Corning, Fisher Scientific #3701). Reactions were incubated at room temperature, with absorbance at 575 nm and 660 nm recorded simultaneously every hour for over 20 h using a PowerWave plate reader. Background was subtracted from both readings, and OD_575_ was normalized to OD_660_ per well. Curves were fit by nonlinear regression using a Michaelis–Menten-type model in GraphPad Prism (version 11) to determine K_50_ values.

### 2.7 Envelope permeabilization assay

To determine whether the identified hit compounds increase envelope permeability in *E. coli* MG1655, CPRG hydrolysis was measured across a chloramphenicol concentration titration, alone or combined with hit compounds. Chloramphenicol was included in all treatments to inhibit *de novo* protein synthesis, ensuring that CPRG hydrolysis reflected outer membrane permeabilization of existing β-galactosidase rather than newly induced enzyme. Overnight culture was diluted 1:50 in sterile LB supplemented with [0.1 mM (100 μM)] IPTG and incubated at 37 °C, 250 rpm until OD_600_ reached 0.4 (mid-log phase, ~1.5–2 h). A compound dilution plate was prepared with a 2-fold chloramphenicol titration spanning 10 concentrations, from [0.62 mM (620 μM)] to [0.0012 mM (1.2 μM)], tested alone or with a hit compound at a fixed concentration. Culture was supplemented with CPRG to 40 mg/L (40 μg/mL), dispensed into a 96-well plate (159 μL/well) for duplicate transfer to a 384-well plate, then the compound was added. Positive control (ampicillin, a known outer membrane permeabilizer), negative/baseline control (LB + *E. coli* + CPRG), and a cell-free background blank (LB + CPRG, no bacteria) were included on each plate. OD_575_ (CPRG hydrolysis, permeabilization) and OD_660_ (growth) were recorded simultaneously every hour for 20 h using a PowerWave plate reader. Abs_575_ was normalized to OD_660_, and normalized CPRG hydrolysis was plotted against chloramphenicol concentration; dose–response curves were fit using GraphPad Prism, version 11 (nonlinear regression, sigmoidal dose–response model).

### 2.8 Minimal inhibitory concentration (MIC) and microscopic assays

MIC and phenotypic assays were performed for isorotenone, suloctidil, and alexidine against *E. coli* K12 MG1655 and *P. aeruginosa* PAO1. MICs were determined in LB broth (1% NaCl) at varying compound concentrations. Cultures were initiated with bacterial suspensions at 2 × 10^6^ CFU/mL and incubated at 37 °C for 18 h. Bacterial growth was monitored by measuring OD_600_ every 30 min. For phenotypic and microscopic analyses, single colonies were grown overnight in 6 mL LB at 37 °C with agitation, diluted to 0.02 (OD_600_) in fresh LB, and transferred to 250 mL flasks with 25 mL LB. Cultures were incubated at 37 °C, 250 rpm, and monitored by OD_600_. At mid-log phase (OD_600_ ≈ 0.5), 3 μL was spotted on 0.8% agarose pads for imaging. Phase-contrast images were acquired using a Nikon Eclipse E600 microscope (100 × phase-contrast, oil-immersion objective) and DS-Ri2 camera.

### 2.9 DAPI/FITC/CTC triple-staining confocal microscopy

*E. coli* MG1655 was grown at 37 °C in LB supplemented with 0.1 mM IPTG to an OD₆₀₀ of 1 (~10^9^ CFU/mL). Cells were pelleted, washed, and resuspended in 10 mM sodium phosphate buffer (PBS, pH 7.4). Vancomycin [final concentration 0.2 mM (200 μM)] was added to one aliquot, and the mixture was incubated for 60 min at 30 °C; an untreated aliquot served as a control. Cells were then washed, resuspended in CTC (5-cyano-2,3-ditolyl tetrazolium chloride; 10 mM in PBS), and incubated for 90 min at 37 °C with agitation (150 rpm) to assess metabolic viability. Cells were transferred onto poly-L-lysine-coated glass slides and incubated at 30 °C for 45 min to allow adhesion, followed by three PBS washes and three rapid deionized-water rinses. Slides were stained with DAPI (4′,6-diamidino-2-phenylindole; 10 μg/mL in PBS) for 30 min at 30 °C to label all cells irrespective of viability, rinsed, then stained with FITC (fluorescein isothiocyanate; 6 mg/mL stock in acetone, diluted in PBS) for 30 min at 30 °C to detect membrane permeabilization, followed by a final wash. Slides were imaged by confocal fluorescence microscopy.

## 3 Results

### 3.1 Optimization and development of the LacZ/CPRG envelope integrity assay

We established the LacZ/CPRG assay as a functional readout of GN envelope permeability in a 96-well microplate format (100 μL reaction volume). CPRG is excluded by an intact envelope but enters the cytoplasm upon membrane disruption, where it is hydrolyzed by β-galactosidase (LacZ) to produce a red chromogenic product detectable at 575 nm. CPRG titration showed a concentration-dependent increase in signal, defined by nonlinear regression analysis (Figure 1Bi). The apparent half-maximal response concentration was 25 mg/L CPRG, while 40 mg/L CPRG (40 μg/mL) provided optimal signal intensity and dynamic range for detecting envelope perturbation. Signal stability was maintained for over 3 h. Activity was detected exclusively in envelope-defective mutants, whereas wild-type cells remained at baseline, consistent with intact envelope exclusion. Representative microplate readouts showed clear visual separation between conditions (Figure 1Bii).

Moreover, we evaluated assay performance using ampicillin as a reference envelope-targeting antibiotic under identical conditions. Ampicillin induced a clear concentration-dependent response (Figure 1Ci), with an apparent half-maximal response concentration of 21 mg/L. Representative plate readouts showed progressive color development (Figure 1Cii). Maximal and reproducible responses were observed with 40 mg/L ampicillin in combination with 40 mg/L CPRG.

The assay was further evaluated in pathogenic *E. coli* strains with intact LPS to assess applicability beyond the O-antigen-deficient MG1655 background (Figure 1D). This phenotypic assay detects GN envelope defects through extracellular CPRG hydrolysis by LacZ, generating a positive signal upon increased permeability or cell lysis. Under optimized conditions, ampicillin (3.71 mg/L; 0.01 mM) produced a positive CPRG response in all strains tested. EHEC showed the highest sensitivity with elevated background signal, whereas UPEC exhibited the weakest response.

After assay optimization, a panel of small molecules was evaluated for effects on envelope integrity. Several compounds induced robust color development in mutants, indicating envelope compromise, including agents that inhibit cell division, promote lysis, or increase membrane permeability and facilitate intracellular uptake of other antimicrobials. Chloramphenicol, a protein synthesis inhibitor, was included as a non–envelope-targeting comparator and did not induce detectable CPRG signal, whereas ampicillin produced a robust response at 40 mg/L CPRG, supporting envelope-dependent signal generation (Figure 1E).

### 3.2 Miniaturization, assay robustness, and statistical validation

To enable high-throughput screening, the LacZ/CPRG assay was systematically optimized for reaction volume and format compatibility across 96- and 384-well platforms using reference antibiotics. To evaluate the effect of miniaturization, the colorimetric CPRG response was assessed across reaction volumes of 200, 100, and 50 μL in the presence of ampicillin, LB + *E. coli* (negative control), chloramphenicol, and LB medium. Progressive reductions in reaction volume resulted in improved signal consistency, with 50 μL providing optimal performance for assay miniaturization. Under optimized conditions, ampicillin produced a robust and reproducible response, with a fourfold increase in absorbance units relative to chloramphenicol and a threefold increase over the LB + *E. coli* (negative control) (Figure 2A). Time-course analysis showed that signal accumulation plateaued after approximately 90 min, with 2 h sufficient to reach near-maximal signal and 3 h providing stable endpoint measurements (Figure 2B). Miniaturizing the assay from 100 μL (96-well format) to 50 μL (384-well format) did not compromise assay performance. Although reduced reaction volumes slightly slowed color development at lower antibiotic concentrations, overall assay robustness remained high, with a Z′-factor of 0.9 in the 384-well format.

**Figure 2:**
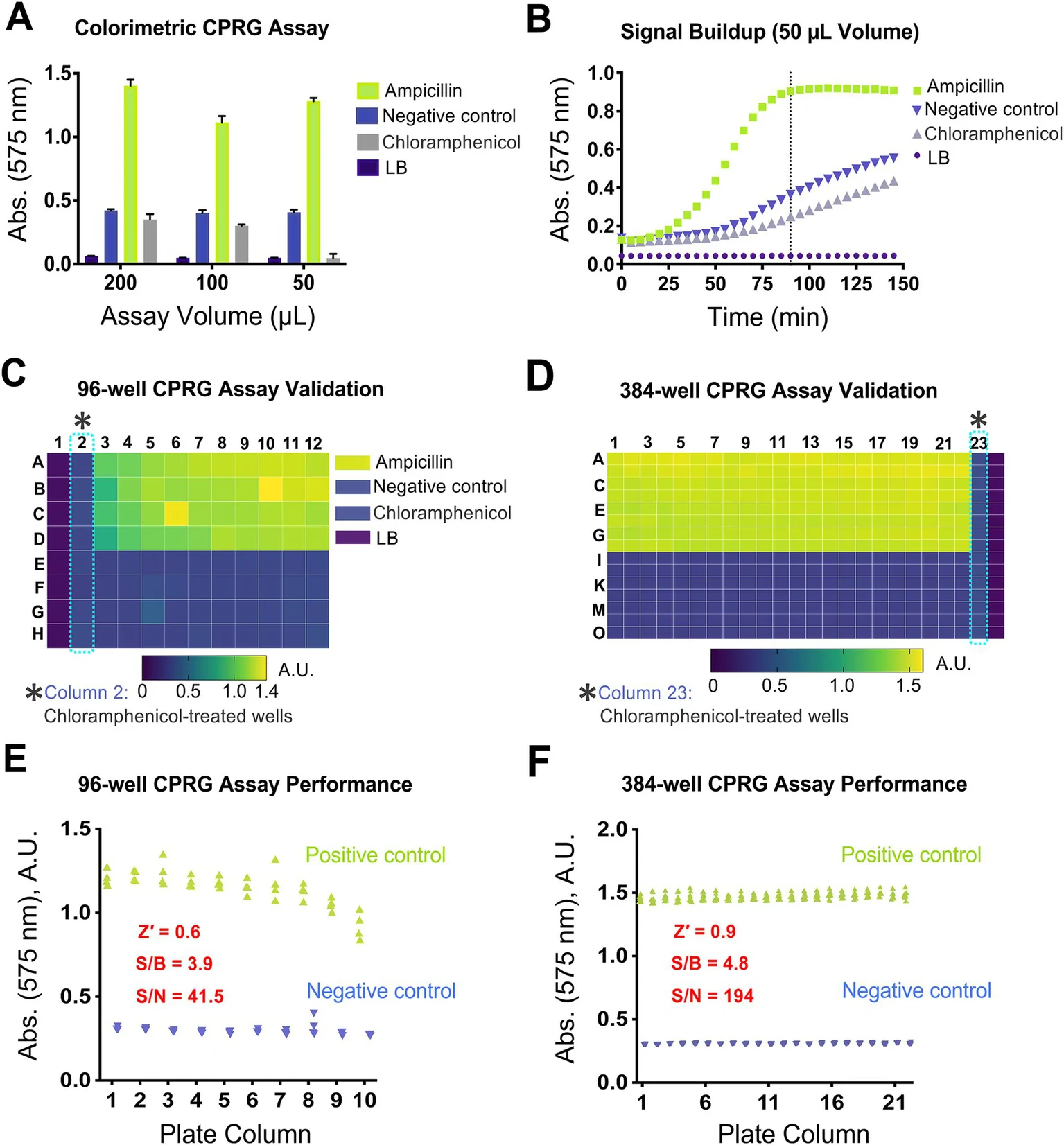
Miniaturization, robustness, and statistical validation of the LacZ/CPRG assay for high-throughput screening. **(A)** CPRG response across 50–200 μL reaction volumes using positive control (ampicillin), negative/baseline control (LB + *E. coli* + CPRG), orthogonal negative control (chloramphenicol), and a cell-free background blank (LB + CPRG, no bacteria). Miniaturization to 50 μL improved signal consistency, with ampicillin showing ~4-fold higher signal than chloramphenicol and ~3-fold higher signal than the negative control. **(B)** Time-course of CPRG signal development (50 μL format) showing a progressive increase with plateau after ~90 min; 2 h incubation was sufficient for near-maximal signal, while 3 h provided a stable endpoint measurement. **(C,D)** Heatmaps of replicate measurements in **(C)** 96-well and **(D)** 384-well formats. Color intensity represents CPRG absorbance at 575 nm (0–1.5 absorbance units, A. U.), ranging from the background blank (LB + CPRG) to maximal signal (ampicillin). Corresponding quantitative data and raw plate images are provided in Supplementary Figures S6, S7. **(E)** The 96-well LacZ/CPRG assay demonstrated robust performance, based on the positive and negative controls (ampicillin-treated and untreated *E. coli*, respectively), as shown by the Z′-factor, S/B, and S/N values. **(F)** Miniaturization to a 384-well format improved assay performance, as shown by these metrics using the same controls.

To visualize assay performance and well-to-well reproducibility, heatmaps were generated for both 96-well and 384-well formats (Figures 2C,D). The heatmaps display replicate measurements for each well based on CPRG absorbance values measured at 575 nm for ampicillin (positive control), LB + *E. coli* (negative control), chloramphenicol (orthogonal negative control), and LB medium alone. Corresponding quantitative analyses, together with the original 96-well and 384-well plate images, are provided in the Supplementary Figures S6, S7.

Comparison of assay formats demonstrated improved performance upon miniaturization. In the 96-well format, ampicillin yielded a Z′-factor of 0.6, with signal-to-background (S/B) and signal-to-noise (S/N) ratios of 3.9 and 41.5, respectively (Figure 2E). Transition to the 384-well format further improved performance, increasing the Z′-factor to 0.9, the S/B ratio to 4.8, and the S/N ratio to 194 (Figure 2F).

Z′-factor analysis confirmed that both formats meet accepted criteria for high-throughput screening, with values above 0.5 indicating acceptable assay performance and values approaching 1.0 reflecting excellent assay quality (Zhang et al., 1999).

To further optimize assay performance, extended overnight incubation with 40 mg/L CPRG increased background signal without improving Z′-factor, S/N, or S/B values, whereas reducing CPRG concentration to 20 mg/L slightly reduced baseline signal but did not improve overall HTS performance (Table 1). The assay performed consistently and reproducibly across independent experiments and technical replicates, supporting its robustness for high-throughput screening applications.

### 3.3 Automation using the LOPAC^1280^ library

With assay robustness validated, we proceeded to automated high-throughput screening (HTS), testing compounds in a single-point format. The LOPAC^1280^ library of biologically active compounds was screened to assess automation specificity and sensitivity after 3 h and overnight incubation at room temperature. The assay showed consistent performance based on control-derived quality metrics (Z’-factor 0.6, S/N 17, S/B 2.6). It identified five hits at 3 h (Supplementary Figure S8A) and eleven after overnight incubation (Supplementary Figure S8B), using a cut-off of ≥25% increase in normalized CPRG signal relative to the negative/baseline control, corresponding to 3 SD above the plate mean (Table 2). Ten overlapped with the KD_2_ screen (Table 3); trimethoprim was uniquely identified in LOPAC. Eight compounds affected cell envelope synthesis; trimethoprim, disulfiram, and minocycline hydrochloride did not but may have indirect effects on bacterial cells. Trimethoprim inhibits dihydrofolate reductase, impairing DNA synthesis (Brogden et al., 1982). Disulfiram, used for alcohol dependence, also shows potential against Lyme disease, cancer, and bacterial and parasitic infections (Liegner, 2019). Minocycline hydrochloride, a semi-synthetic tetracycline, blocks protein synthesis in GP and GN bacteria and exhibits additional non-antibiotic properties (Garrido-Mesa et al., 2013). Although these compounds do not directly target the cell wall, they may indirectly alter envelope permeability and influence the CPRG assay by affecting metabolism, DNA replication, or protein synthesis, thereby modifying β-galactosidase activity and CPRG hydrolysis.

**Table 2:** Active compounds identified in the LOPAC1280 screen (11 actives), showing >25% increase in OD absorbance relative to the negative control.

Plate ID	Well ID	Compound name	Function	Group
1	F12	Ceftriaxone sodium	Cell wall synthesis	Cephalosporin, 3rd gen
1	F14	Cefmetazole sodium	Cell wall synthesis	Cephalosporin, 2nd gen
1	H10	Cefazolin sodium	Cell wall synthesis	Cephalosporin, 1st gen
1	H18	Cefotaxime sodium	Cell wall synthesis	Cephalosporin, 3rd gen
1	H20	Cephapirin sodium	Cell wall synthesis	Cephalosporin, 1st gen
1	J8	Cephalothin sodium	Cell wall synthesis	Cephalosporin, 1st gen
1	D16	Cefaclor	Cell wall synthesis	Cephalosporin, 2nd gen
4	N16	Vancomycin	Cell wall synthesis	Glycopeptides antibio.
3	B5	Minocycline hydrochloride	Protein synthesis, Cell cycle	Tetracycline/Polyketide
4	J9	Tetraethyl thiuram disulfide	Alcohol dehydrogenase inhibitor	Organosulfur compounds
4	P4	Trimethoprim	Dihydrofolate reductase	Diaminopyrimidine

**Table 3:** Active compounds identified in the KD_2_ library screen (72 actives: 42 at 3 h, 30 after overnight incubation) showing >25% increase in OD absorbance relative to the negative control.

Well ID*	Compound name	Function	Group	3 h OD, %	Overnight OD, %	KD/LOPAC
**F12**	Ceftriaxone	Cell wall synthesis	Cephalosporin, 3rd gen	271	135	X
F14	Cefmetazole sodium	Cell wall synthesis	Cephalosporin, 2nd gen	101	168	X
**C14**	Cefazolin sodium	Cell wall synthesis	Cephalosporin, 1st gen	200	145	X
**H18**	Cefotaxime sodium	Cell wall synthesis	Cephalosporin, 3rd gen	253	160	X
**H20**	Cephapirin sodium	Cell wall synthesis	Cephalosporin, 1st gen	142	182	X
**O19**	Cephalothin sodium	Cell wall synthesis	Cephalosporin, 1st gen	177	156	X
D16	Cefaclor	Cell wall synthesis	Cephalosporin, 2nd gen	100	168	X
N16	Vancomycin	Cell wall synthesis	Glycopeptides	104	172	X
B5	Minocycline-HCl	Protein synthesis	Tetracycline/Polyketide	112	136	X
J9	Tetraethylthiuram-DS	Alcohol dehydrogenase	Organosulfur	84	164	X
K19	Alexidine hydrochloride	Cell membrane	Biguanide	114	161	
G10	Ampicillin trihydrate	Cell wall synthesis	Penicillin class	104	128	
F3	Azlocillin sodium	Cell wall synthesis	Ureidopenicillin	101	129	
**F11**	Aztreonam	Cell wall synthesis	Monobactams	165	147	
I12	Brazilein	Cell membrane	Anthraquinone	102	127	
B11	Cefaclor	Cell wall synthesis	Cephalosporin, 2nd gen	110	159	
**E16**	Cefamandole sodium	Cell wall synthesis	Cephalosporin, 2nd gen	183	145	
**F13**	Cefamandole sodium	Cell wall synthesis	Cephalosporin, 2nd gen	154	150	
**H22**	Cefazolin	Cell wall synthesis	Cephalosporin, 1st gen	202	158	
**H10**	Cefazolin sodium	Cell wall synthesis	Cephalosporin, 1st gen	197	167	
**M9**	Cefdinir	Cell wall synthesis	Cephalosporin, 3rd gen	181	150	
**O18**	Cefepime hydrochloride	Cell wall synthesis	Cephalosporin, 4th gen	234	134	
**M6**	Cefixime	Cell wall synthesis	Cephalosporin, 3rd gen	232	134	
**K21**	Cefmetazole sodium	Cell wall synthesis	Cephalosporin, 2nd gen	228	129	
F15	Cefmetazole sodium	Cell wall synthesis	Cephalosporin, 2nd gen	110	155	
**K14**	Cefonicid	Cell wall synthesis	Cephalosporin, 2nd gen	172	149	
**F21**	Cefoperazone sodium	Cell wall synthesis	Cephalosporin, 3rd gen	183	150	
**A15**	Cefoperazone dihydrate	Cell wall synthesis	Cephalosporin, 3rd gen	238	118	
**M22**	Ceforanide	Cell wall synthesis	Cephalosporin, 2nd gen	150	140	
**K20**	Cefotaxime sodium	Cell wall synthesis	Cephalosporin, 3rd gen	230	132	
**J4**	Cefotaxime	Cell wall synthesis	Cephalosporin, 3rd gen	226	123	
**O8**	Cefotetan	Cell wall synthesis	Cephalosporin, 2nd gen	232	129	
**B5**	Cefotiam hydrochloride	Cell wall synthesis	Cephalosporin, 2nd gen	205	105	
**H5**	Cefoxitin sodium	Cell wall synthesis	Cephalosporin, 2nd gen	225	137	
F5	Cefoxitin sodium	Cell wall synthesis	Cephalosporin, 2nd gen	110	156	
**B19**	Ceftazidime 5-H	Cell wall synthesis	Cephalosporin, 3rd gen	155	141	
**C13**	Ceftazidime	Cell wall synthesis	Cephalosporin, 3rd gen	137	171	
**M7**	Ceftibuten	Cell wall synthesis	Cephalosporin, 3rd gen	197	149	
**N11**	Ceftriaxone	Cell wall synthesis	Cephalosporin, 3rd gen	229	137	
**O21**	Cefuroxime sodium	Cell wall synthesis	Cephalosporin, 2nd gen	200	141	
**F9**	Cefuroxime	Cell wall synthesis	Cephalosporin, 2nd gen	182	148	
**J8**	Cephalothin sodium	Cell wall synthesis	Cephalosporin, 1st gen	163	176	
**J6**	Cephalothin	Cell wall synthesis	Cephalosporin, 1st gen	183	164	
**J8**	Cephapirin	Cell wall synthesis	Cephalosporin, 1st gen	146	166	
**D15**	Chicago sky blue 6B	Alter membrane	Stain reagent	131	120	
G8	Ciprofloxacin-HCl	DNA replication	Fluoroquinolone, 2nd gen	100	129	
I18	Ciprofloxacin	DNA replication	Fluoroquinolone, 2nd gen	107	133	
G11	Colistin sulfate	Cell membrane	Polymyxin E sulfate	113	167	
K19	**DOCA	Hits non-antibiotic	Mineralocorticoid	100	109	
B17	Disulfiram	Thiol disruption	Antiprotozoal agents	85	142	
C16	Disulfiram	Thiol disruption	Antiprotozoal agents	90	148	
**M14**	Faropenem (Fropenem)	Cell wall synthesis	β-lactam penem	195	102	
**H15**	Gentian violet	Membrane/Nucleic	Antiseptic dye	138	121	
**J11**	Hetacillin potassium	Cell wall lysis	β-lactam penicillin	147	143	
**E16**	Isorotenone	Cellular integrity	Terpenoids	159	164	
M14	Meropenem	Cell wall lysis	β-lactam carbapenem	97	159	
O14	Meropenem	Cell wall lysis	β-lactam carbapenem	101	143	
P11	Metampicillin sodium	Cell wall inhibitor	β-lactam penicillin	97	152	
**E18**	Monensin sodium	Osmotic regulation	Ionophore	182	110	
**D19**	Moxalactam disodium	Cell wall synthesis	Cephalosporin subclass	217	139	
**M22**	Parbendazole	Cell division	Benzimidazole	161	115	
A22	Pazufloxacin	DNA/Cell division	Fluoroquinolone	91	125	
N20	Piperacillin sodium	Cell wall synthesis	β-lactam penicillin	101	155	
**G12**	Piperacillin sodium	Cell wall synthesis	β-lactam penicillin	151	151	
C8	Pivmecillinam-HCl	Cell wall synthesis	β-lactam penicillin	103	155	
I12	Polymyxin B	Cell membrane	Cyclic polypeptide	101	155	
**K10**	Sisomicin sulfate	Protein synthesis	Aminoglycoside	225	124	
M22	Suloctidil	Vasodilators	Non-antibiotic	115	165	
I21	Talampicillin-HCl	Cell wall synthesis	Aminopenicillins	99	141	
H6	Thiram	Protein/Respiratory	Organosulfur	88	160	
O10	Vancomycin	Cell wall synthesis	Glycopeptide antibiotic	100	132	
E18	Vancomycin	Cell wall synthesis	Glycopeptide antibiotic	110	154	

Ten of the 11 LOPAC^1280^ hits were also represented in KD_2_. *Bold Well IDs indicate the 42 hits identified at the 3-h incubation timepoint (Figure 3). **Deoxycorticosterone acetate.

### 3.4 Automation by screening the KD_2_ library in HTS

Primary screening was performed in a single-point format per compound. To expand screening coverage, 3,840 compounds from the KD_2_ library were screened, and their effects on CPRG signal distribution were plotted and analyzed (Figure 3). Screen performance was evaluated using standard HTS quality-control metrics derived from control wells, with favorable Z’, S/B, and S/N values (Zhang et al., 1999) (Supplementary Figure S9). Seventy-two envelope-targeting hits showing ≥25% increase in normalized CPRG signal relative to the negative/baseline control were identified—42 at 3 h and an additional 30 after overnight incubation (Figure 3; Table 3). Among the KD_2_ hits, 90% were known antibiotics. β-lactams accounted for 80% of hits (57 compounds, 1.2% of the library); 60% of these were unique compounds, with the remainder representing duplicates. The LOPAC1280 screen identified 11 active compounds, 10 of which were confirmed in the KD_2_ screen (Table 2).

**Figure 3:**
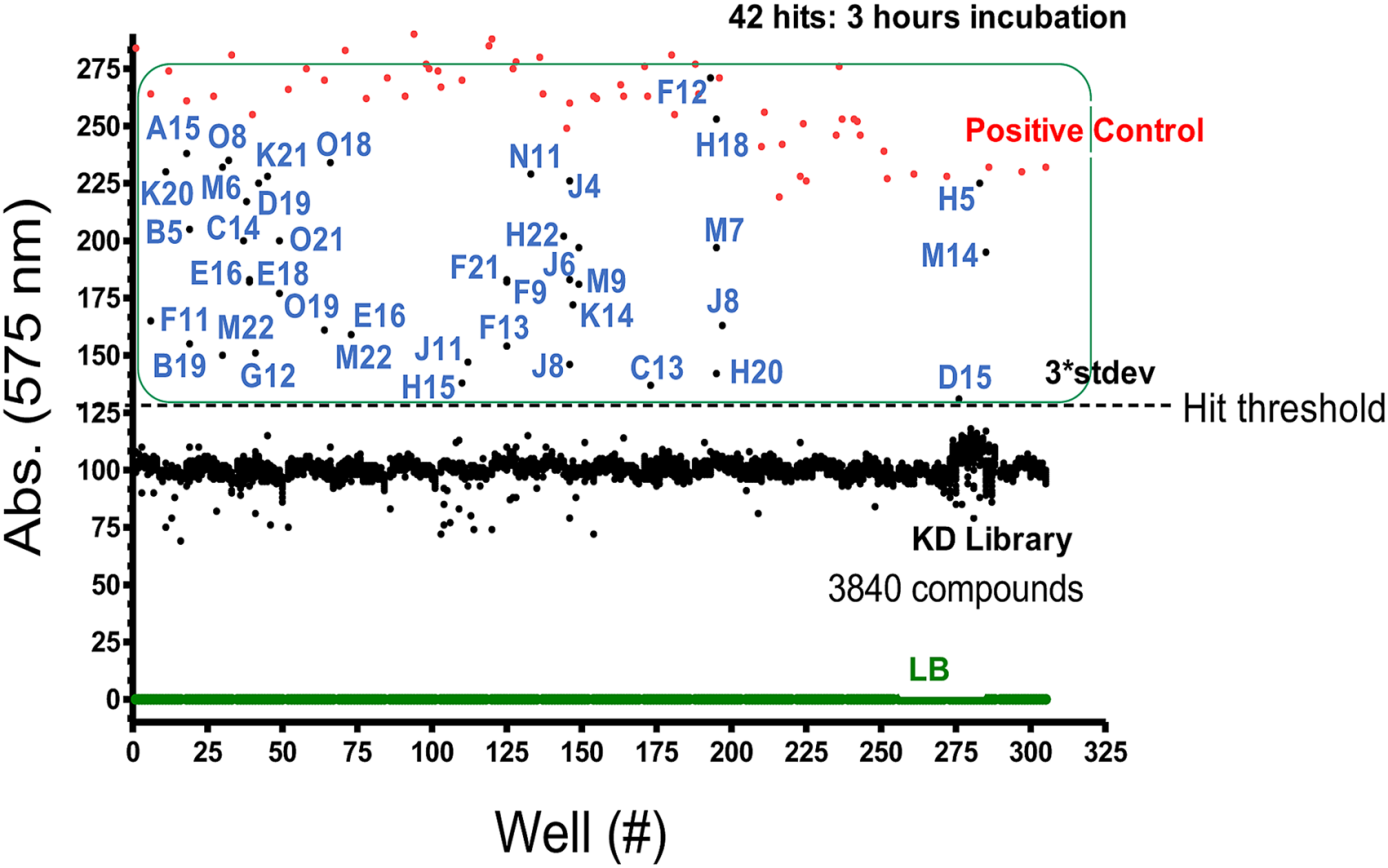
High-throughput screening of the KD_2_ library identifies active hits. A subset of 3,840 compounds from the KD_2_ library (4,761 compounds total) was screened against *E. coli*. Distribution of OD_575_ values for all screened compounds, normalized to the background blank (LB + CPRG, no bacteria; 0%) and negative control (*E. coli* + CPRG; 100%); each point represents a single-point well measurement. Compounds producing an OD_575_ signal ≥25% above the negative control (≥125% normalized OD_575_; corresponding to 3 SD above the negative control mean) were considered hits, as indicated by the dashed hit-threshold line. The 42 compounds identified after 3 h are shown and labeled. Ampicillin, the positive control (red dots), produced a strong signal increase relative to the background (~240%) across all assay plates. An additional 30 compounds were identified after overnight incubation, for a total of 72 hits; full details are provided in Table 3. Quality-control metrics (Z′-factor, S/N, and S/B) are presented in Supplementary Figure S9.

### 3.5 Confirmation of 72 envelope-targeting actives by absorbance assays

Following CPRG assay screening of the LOPAC and KD_2_ libraries, 72 compounds (Table 3) were selected for re-evaluation to validate primary screening hits. The assay was performed using independent experimental repeats. Cherry-picking followed the protocol, with 3-h and overnight incubations. Absorbance at 575_nm_ was monitored for 11 h to classify leakage rates (slow, moderate, and rapid). Leakage rates were defined based on the time at which the A_575_ nm signal exceeded the DMSO baseline. Rapid leakage was characterized by an early increase in CPRG signal A_575_, moderate leakage by an intermediate increase, and slow leakage by a delayed increase during the incubation period. Thirteen compounds were “slow leakers,” nine “moderate,” and the rest exhibited rapid action. Seven compounds failed to confirm, and eight compounds inhibited growth in MIC assays without detectable changes in CPRG signal (Supplementary Figure S10). Consistent with Section 3.4, the majority of confirmed hits were β-lactams, increasing absorbance ≥25% at 0.0063 mM (6.3 μM); the remaining hits included four non-antibiotic compounds—isorotenone, alexidine, suloctidil, and thiram—which also increased absorbance ≥25%, confirming permeabilization (Table 3).

### 3.6 Concentration–response and permeabilization assessment

To mitigate fluorescence and light-detection artifacts common in HTS (Simeonov et al., 2008), primary hits were retested in concentration–response assays to confirm putative actives. A pilot experiment using four hit compounds (isorotenone, alexidine, suloctidil, and thiram) at a fixed concentration of [0.025 mM (25 μM)] showed no difference in normalized CPRG signal between initial bacterial densities of OD_600_ = 0.1 and 0.4, or between 3 h and 5 h incubation, supporting OD_600_ = 0.1 as the working density for subsequent titration experiments. All four compounds, including thiram, induced measurable permeabilization in this pilot; however, thiram was excluded from further testing due to its fungicidal use. We then generated concentration–response curves for the remaining six compounds (vancomycin, cefaclor, cefsulodin, isorotenone, suloctidil, and alexidine) to determine the relationship between compound concentration and permeabilization. OD_575_ data were normalized to OD_660_ to account for concentration-dependent effects on bacterial growth. The permeabilization constant (K_50_) was defined as the concentration eliciting 50% of the maximal normalized CPRG signal. Assays were conducted at room temperature; DMSO served as the vehicle control. All six compounds exhibited concentration-dependent permeabilization, with K_50_ values spanning nearly three orders of magnitude (Figure 4; Supplementary Table S1). Alexidine, a non-antibiotic, was the most potent -roughly 15-fold more active than vancomycin and 400-fold more active than isorotenone.

**Figure 4:**
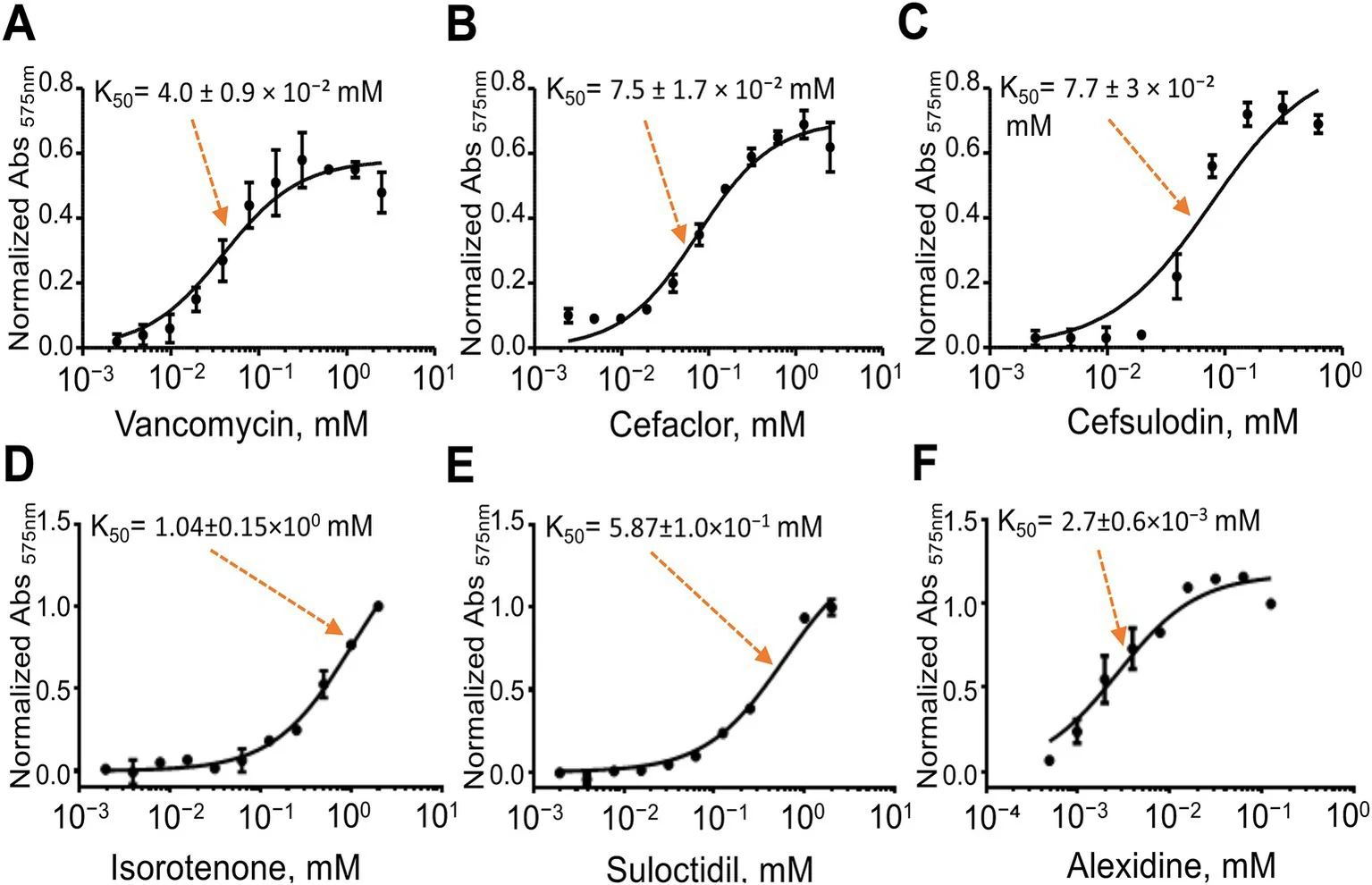
Permeabilization curves determining *K*_50_ for six compounds identified in the primary KD_2_ library screen. *K*_50_ values, calculated by nonlinear regression, represent the concentration at which each compound induced 50% of maximal envelope permeabilization (arrow). The *X*-axis shows compound concentration (mM); the *Y*-axis shows absorbance at 575 nm (CPRG product), normalized to OD_660_. **(A)** Vancomycin, **(B)** Cefaclor, **(C)** Cefsulodin, **(D)** Isorotenone, **(E)** Suloctidil, and **(F)** Alexidine each exhibit a concentration-dependent sigmoidal response, reflecting dose-dependent envelope membrane permeabilization. Alexidine **(F)** reached *K*_50_ at the lowest concentration among the six compounds tested. Data represent mean ± SD of three independent biological replicates (*n* = 3).

### 3.7 Hit compounds increase envelope permeability in a chloramphenicol-dependent manner

CPRG hydrolysis (normalized Abs_575_) was measured across a chloramphenicol titration ([0.0012–0.62 mM (1.2–620 μM)]) in *E. coli* MG1655, alone or with a hit compound at a fixed concentration. Chloramphenicol alone produced minimal CPRG hydrolysis except at the highest concentrations tested (Figure 5A). Ampicillin [0.05 mM (50 μM)] produced a marked, dose-dependent increase in CPRG hydrolysis with rising chloramphenicol concentration, plateauing at a normalized Abs_575_ of ~1.5–1.6, whereas vancomycin [0.05 mM (50 μM)] did not increase signal above baseline (Figure 5A). Among the compounds in Figure 5B, alexidine [0.002 mM (2 μM)] produced the strongest response, active at markedly lower chloramphenicol concentrations and plateauing near Abs_575_ ≈ 3.0; suloctidil [0.5 mM (500 μM)] also increased CPRG hydrolysis dose-dependently, plateauing at ~1.5–1.6 but at higher chloramphenicol concentrations than alexidine. Isorotenone [0.5 mM (500 μM)] showed no increase above baseline. These results demonstrate that ampicillin, alexidine, and suloctidil selectively increase outer membrane permeability in a chloramphenicol-dependent manner, whereas vancomycin and isorotenone do not, highlighting their potential as adjuvants for combination therapies targeting the GN outer membrane. Data represent mean ± SD of three independent biological replicates (*n* = 3).

**Figure 5:**
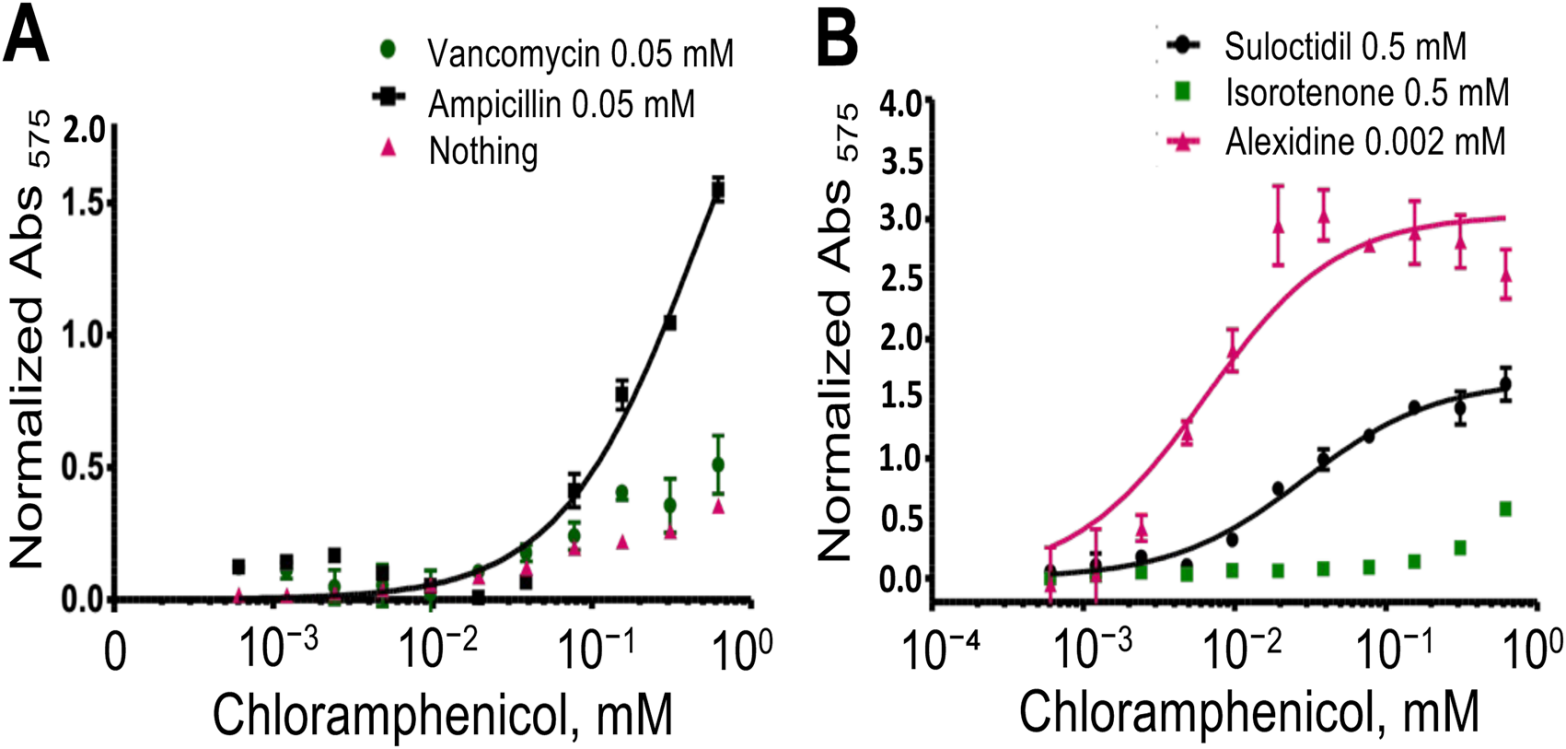
Hit compounds increase cell envelope permeability in *E. coli* across a chloramphenicol concentration titration. CPRG hydrolysis (normalized Abs_575_) was measured in *E. coli* MG1655 across a 2-fold chloramphenicol titration (0.0012–0.62 mM), alone or with a hit compound at a fixed concentration, with OD_575_ and OD_660_ recorded simultaneously every hour for 20 h. **(A)** Ampicillin (0.05 mM) produced a dose-dependent increase in CPRG hydrolysis, while vancomycin (0.05 mM) and chloramphenicol alone (nothing) showed minimal response. **(B)** Alexidine (0.002 mM) and suloctidil (0.5 mM) increased CPRG hydrolysis dose-dependently, with alexidine active at substantially lower chloramphenicol concentrations; isorotenone (0.5 mM) showed no increase above baseline. Data represent mean ± SD of three independent biological replicates (*n* = 3).

### 3.8 MIC and microscopy assays

To validate antibacterial activity and morphological effects, MIC assays confirmed the antibacterial activity of alexidine, suloctidil, and isorotenone against *E. coli* and *P. aeruginosa*. MIC values were determined in independent biological replicates (*n* = 3). Microscopy revealed corresponding morphological changes (Figures 6A,B). Alexidine was the most potent, with MICs of 0.004 mM (4 μM) for *E. coli* and 0.015 mM (15 μM) for *P. aeruginosa* (Figure 6C). At sub-MIC concentrations, alexidine induced filamentation (Figures 6D,E), reflecting division defects associated with envelope disruption. Suloctidil showed MICs of 0.125 mM (125 μM) for *E. coli* and 0.5 mM (500 μM) for *P. aeruginosa* (Figure 6F). Sub-MIC exposure led to a more spherical shape (Figures 6G,H), with lysis below the MIC suggesting compromised peptidoglycan integrity and osmotic stability. Isorotenone had an MIC of 0.25 mM (250 μM) for both species (Figure 6I). Like suloctidil, it induced spherical morphology at sub-MIC levels, with lysis below the MIC (Figures 6J,K). Overall, these results indicate membrane disruption that weakens cell wall integrity and osmotic resistance, providing a functional readout of envelope integrity complementary to established permeability assays.

**Figure 6:**
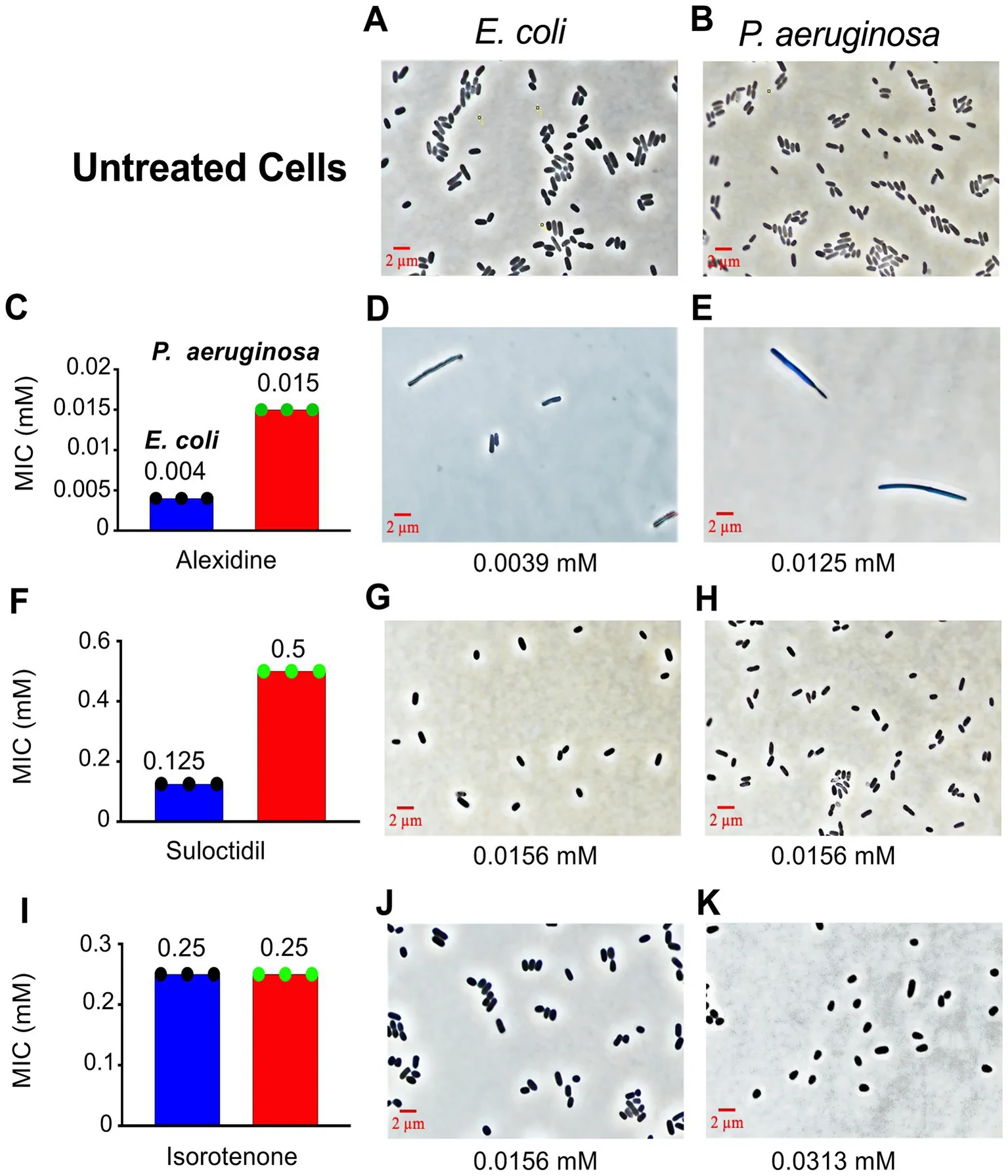
MIC and morphological effects of alexidine, suloctidil, and isorotenone in *E. coli* and *P. aeruginosa*. **(A,B)** Microscopy images comparing the morphology of untreated bacterial cells. **(C)** Alexidine exhibited the lowest MICs: 0.004 mM (4 μM) for *E. coli* and 0.015 mM (15 μM) for *P. aeruginosa*. **(D,E)** Sub-MIC alexidine (0.0039 and 0.0125 mM) induced filamentous morphology. **(F)** Suloctidil had MICs of 0.125 mM (125 μM) for *E. coli* and 0.5 mM (500 μM) for *P. aeruginosa*. **(G,H)** Treated cells became spherical and showed lysis below the MIC (0.0156 mM). **(I)** Isorotenone had an MIC of 0.25 mM (250 μM) for both species. **(J,K)** Sub-MIC concentrations (0.0156 and 0.0313 mM) caused spherical morphology and lysis. These phenotypes suggest membrane and cell wall disruption. MICs were determined in independent biological replicates (*n* = 3; *SD* = 0). Images were acquired using a 100 × phase-contrast, oil-immersion objective and represent independent experiments.

### 3.9 Validation of a confocal microscopy-based secondary assay using vancomycin

To complement the CPRG-based permeabilization assay with an orthogonal, single-cell-resolution method, a DAPI/FITC/CTC triple-staining confocal microscopy approach was developed using vancomycin, a compound with well-characterized envelope-disrupting activity. DAPI labeled all cells irrespective of viability, FITC labeled cells with compromised membrane integrity, and CTC labeled metabolically viable cells (Supplementary Figure S11). DAPI staining was comparable between control and vancomycin-treated cells, confirming similar total cell counts. Representative images showed a qualitative increase in FITC staining in vancomycin-treated cells relative to controls, consistent with membrane permeabilization, alongside a modest, qualitative reduction in CTC staining, suggesting a partial decrease in viability. These observations support the feasibility of triple-staining confocal microscopy as a complementary, single-cell method for future validation of envelope-permeabilizing compounds.

## 4 Discussion

This study introduces a validated, sensitive phenotypic assay in liquid media to identify candidate molecules targeting the GN bacterial envelope. The LacZ/CPRG assay, optimized for high-throughput screening (HTS), detects envelope permeability defects by measuring OD at 575 nm. Collectively, these findings indicate that the LacZ/CPRG assay effectively captures perturbations in envelope integrity, providing mechanistic insight into bacterial susceptibility while offering complementary information to traditional permeability assays without requiring layer-specific measurements. These findings provide a valuable tool for antibiotic discovery and may help identify novel antibacterial agents.

The assay enables efficient high-throughput screening of compound libraries, as supported by favorable Z′-factor, S/B, and S/N performance metrics across control conditions. Optimization for 384-well plates, with compatibility for 1,536-well HTS (Mayr and Bojanic, 2009) may further extend its scalability.

Screening of the KD_2_ library (3,840 compounds) identified 72 active compounds: 42 hits after 3 h incubation (≥25% increase in normalized CPRG signal relative to the negative control) and 30 additional hits after overnight incubation. Notably, 90% of hits were known antibiotics, with β-lactams comprising 80% of hits (57 compounds, 1.2% of the library), 60% of which were unique. Identified antibiotics included vancomycin, cefaclor, and cefsulodin, alongside non-antibiotic compounds such as alexidine, suloctidil, and isorotenone, which were further validated in concentration-response assays confirming dose-dependent permeabilization.

Interestingly, vancomycin was consistently detected in our screen, despite its established inactivity against GN bacteria due to the LPS-rich outer membrane, which blocks access to periplasmic targets (Boll et al., 2016). The LacZ/CPRG assay reports envelope permeability rather than bactericidal activity; therefore, the observed signal likely reflects subtle or transient perturbations in membrane integrity that permit CPRG entry without implying antibacterial activity or growth inhibition. Although MG1655 (rfb-50) lacks O-antigen, this deficiency alone does not confer vancomycin susceptibility, consistent with its limited impact on permeability to specific compounds such as bile salts (Qin et al., 2023). These results indicate that the LacZ/CPRG assay functions as a sensitive reporter of envelope destabilization rather than evidence of canonical vancomycin activity in GN cells. This observation raises mechanistic questions about envelope permeability changes and the conditions that may permit entry of normally excluded compounds, warranting further mechanistic investigation.

Among the identified compounds, alexidine emerged as the most potent, demonstrating strong envelope-disrupting activity at low concentrations. This bisbiguanide antiseptic destabilizes bacterial membranes by binding to negatively charged phospholipids, enhancing membrane permeability, and inducing cell lysis, resulting in irregular cell morphology and bactericidal effects (Zorko and Jerala, 2008). Our results suggest that alexidine is a promising candidate for targeting GN bacterial envelopes, with demonstrated antibacterial efficacy against *E. coli* and *P. aeruginosa*. Previously studied for its ability to neutralize lipopolysaccharides (LPS) and lipoteichoic acids (LTA), alexidine is also employed topically as a disinfectant against a broad spectrum of GN and GP bacteria, further supporting its potential as an antibacterial agent. Additionally, alexidine has exhibited strong antifungal activity against both planktonic and biofilm forms of human fungal pathogens (Zorko and Jerala, 2008; Mamouei et al., 2018), as well as *Enterococcus faecalis* in dentin blocks (Kim et al., 2013). However, its cytotoxicity has been assessed in various mammalian cell lines (Kim et al., 2013; Mamouei et al., 2018) and immune cells, such as primary bone marrow-derived macrophages (Mamouei et al., 2018), where it exhibited slightly higher toxicity to macrophages (CC_50_ > 5 mg/L) compared to cell lines (Mamouei et al., 2018). This underscores the need for further assessment of its selectivity and therapeutic index.

In addition to alexidine, suloctidil and isorotenone also induced concentration-dependent increases in normalized CPRG signal consistent with envelope permeabilization. Although isorotenone was substantially less potent, consistent with its high K_50_ (Figure 4; Supplementary Table S1), at the single fixed concentration tested in the chloramphenicol-titration assay (0.5 mM), isorotenone did not increase signal above baseline (Figure 5B), likely reflecting a concentration below its permeabilization threshold. Suloctidil, a peripheral vasodilator, disrupts lipid homeostasis and increases membrane fluidity, inducing bactericidal morphological changes (Amakawa et al., 2020). Isorotenone, a rotenoid compound, inhibits respiration, depletes energy reserves, and potentially alters cell morphology, exhibiting antimicrobial, antifungal, and anticancer properties, although further investigation is warranted.

These compounds induced distinct morphological changes at sub-MIC levels, consistent with envelope and cell wall disruption (Figure 6). Potential mechanisms include membrane destabilization, impaired cell wall maintenance, cytoskeletal disruption, or osmotic imbalance arising from periplasmic membrane–cell wall decoupling, potentially compounded by non-specific stress responses such as membrane remodeling or division defects (van Teeseling et al., 2017; Ojkic et al., 2019; Ojkic et al., 2022). Further analyses, including peptidoglycan profiling, osmotic sensitivity assays, and electron microscopy, are needed to elucidate the primary mode of action.

To assess the functional impact of envelope permeabilization on antibiotic uptake, we measured CPRG hydrolysis across a chloramphenicol concentration gradient, alone or with hit compounds. Ampicillin, alexidine, and suloctidil each caused a concentration-dependent increase in the chloramphenicol-induced CPRG signal, indicating enhanced outer membrane permeability. Alexidine showed the greatest effect, with activity at lower chloramphenicol concentrations and the highest maximal response (Figure 5), consistent with its K_50_ profile (Figure 4). Conversely, vancomycin and isorotenone showed no increase over chloramphenicol alone, consistent with their weak or absent permeabilizing activity (Figure 5). Together, these findings suggest that ampicillin, alexidine, and suloctidil enhance intracellular antibiotic accumulation and may serve as adjuvants for combination therapy against GN pathogens.

To explore permeabilization at single-cell resolution, we developed a DAPI/FITC/CTC confocal microscopy approach in vancomycin-treated *E. coli* and observed a qualitative increase in FITC uptake alongside a modest, qualitative reduction in viability relative to untreated controls (Supplementary Figure S11), consistent with localized membrane permeabilization rather than strong bactericidal activity. As this proof-of-concept was based on representative imaging of vancomycin only, quantitative validation and extension to alexidine, suloctidil, and isorotenone represent important next steps to confirm permeabilization activity and distinguish bactericidal from membrane-permeabilizing-only effects.

While we did not directly benchmark our LacZ/CPRG assay against established permeability methods (e.g., NPN uptake, nitrocefin hydrolysis, PI/SYTOX staining), its favorable Z-factor, strong signal-to-noise, and reproducible detection of envelope-disrupting compounds underscore its value as a robust HTS tool. Although many of the identified compounds are known antibiotics with limited potential for further development, the discovery of novel molecules such as alexidine, suloctidil, and isorotenone highlights the ability of this screening approach to uncover previously unrecognized antibacterial candidates. These compounds’ ability to destabilize the bacterial envelope offers potential for developing new antibiotics or combination therapies. Our HTS platform, which enables rapid, large-scale screening, could play a pivotal role in identifying additional compounds targeting the bacterial envelope—a critical area for overcoming resistance mechanisms in GN pathogens (Delcour, 2009; McKenna, 2013). This study underscores the importance of developing novel strategies focused on the bacterial envelope, especially considering rising antimicrobial resistance (Brown and Wright, 2016).

Compared with other high-throughput antibacterial assays (Sarker et al., 2007; Nayar et al., 2015; Avesar et al., 2017; Paytubi et al., 2017; Syal et al., 2017; Haynes et al., 2018; Kulesa et al., 2018; Pitruzzello et al., 2019), and despite recent advances in diagnostic tools for antibacterial resistance (Miller et al., 2019; World Health Organization, 2019; Park et al., 2019; Vasala et al., 2020; Song and Lei, 2021; Gajic et al., 2022; Hattab et al., 2024; Meulebroeck et al., 2024; Sturm et al., 2024), our LacZ/CPRG assay stands out for its affordability, simplicity, speed, and high specificity for the bacterial cell envelope. Its superior sensitivity makes it invaluable for screening GN and potentially GP pathogens, with significant potential for integration into drug discovery pipelines. By providing an efficient functional assessment of envelope integrity, this platform complements existing screening approaches and allows detection of compounds that may be overlooked by assays focused solely on viability or layer-specific permeabilization, thereby expanding opportunities to identify novel antibacterial agents. This assay may enhance the identification of novel antibiotics—whether by directly killing bacterial cells, blocking cell division, or augmenting the activity of other drugs via membrane permeabilization. Its flexibility also allows screening of larger compound libraries—from 384-well to 1,536-well plates—thereby boosting throughput and expanding the discovery of new drug candidates.

Our study demonstrated the utility of the LacZ/CPRG assay in identifying compounds that target the bacterial envelope, highlighting its value for antibiotic discovery. Consistent with the LacZ/ONPG assay developed in a 96-well format to screen peptides as helper drug candidates that enhanced envelope permeability in *E. coli* (Baker et al., 2016), our platform shows promise for identifying antibiotics suitable for both monotherapy and combination therapy. Amid the growing threat of antibiotic-resistant GN bacteria, this assay represents a critical step in developing effective therapeutics with a reduced risk of resistance evolution. Future efforts, in conjunction with deep learning methods (Gajic et al., 2022; Sturm et al., 2024), will expand screening to include a broader range of chemical libraries, advancing the development of envelope-targeting antibiotics to combat multidrug-resistant pathogens.

## 5 Conclusion

We developed a high-throughput LacZ/CPRG assay that detects compounds disrupting the GN envelopes, identifying both known antibiotics and novel molecules, including alexidine, suloctidil, and isorotenone, with potential for monotherapy or combination therapy. Although direct comparisons with traditional permeability assays were not performed, this approach provides a rapid functional measure of envelope perturbation that may capture activity missed by conventional or layer-specific screens, thereby broadening the scope of antibacterial discovery. Future integration with computational approaches and expanded chemical libraries will accelerate the development of envelope-targeting therapeutics against multidrug-resistant pathogens.

## Data Availability

The original contributions presented in the study are included in the article and its Supplementary material, further inquiries can be directed to the corresponding authors.
