# Supplementary material for: High-throughput LacZ/CPRG assay for identifying Gram-negative envelope-targeting antibiotics
Source: Front Microbiol. 2026 Sep 23;17:1779546. doi: 10.3389/fmicb.2026.1779546 (PMC13642723; doi:10.3389/fmicb.2026.1779546)
Supplement: Supplementary file 1 [file Supplementary_file_1.pdf]

Supplementary data

High-throughput LacZ/CPRG assay for identifying Gram-negative envelope–targeting antibiotics

Running title: LacZ/CPRG high-throughput screen for envelope-disrupting antibiotics

Fardin Ghobakhlou[1*], Larbi Mokhtari[1], Tom A. Pfeifer[2*], and Catherine Paradis-Bleau[1]

[1]Department of Microbiology, Infectiology and Immunology, Faculty of Medicine, Université de Montreal, Quebec, Canada; [2]Biofactorial, Life Sciences Center, University of British Columbia, Vancouver, British Columbia, Canada.

*Corresponding authors:

Email: fardinghobakhlou@gmail.com

Email: tpfeifer@mail.ubc.ca

This file contains supplementary material referenced in the main text, including assay optimization data, validation, high-throughput screening data, confocal microscopy validation, permeability analyses, and supporting constants.

Contents:

Supplementary Figure S1. Plate reader validation.

Supplementary Figure S2. CPRG signal discrimination.

Supplementary Figure S3. CPRG kinetics in 96-well format.

Supplementary Figure S4. CPRG kinetics in 384-well format.

Supplementary Figure S5. Plate preparation and compound transfer protocol.

Supplementary Figure S6. 96-well absorbance data and plate images.

Supplementary Figure S7. 384-well absorbance data and plate images.

Supplementary Figure S8. LOPAC1280 screening results.

Supplementary Figure S9. Quality-control metrics for the KD2 library.

Supplementary Figure S10. Time-course of compound-induced permeability.

Supplementary Figure S11. Validation of DAPI/FITC/CTC confocal microscopy.

Supplementary Table S1. Permeabilization constants (K_{50}) of six selected compounds.

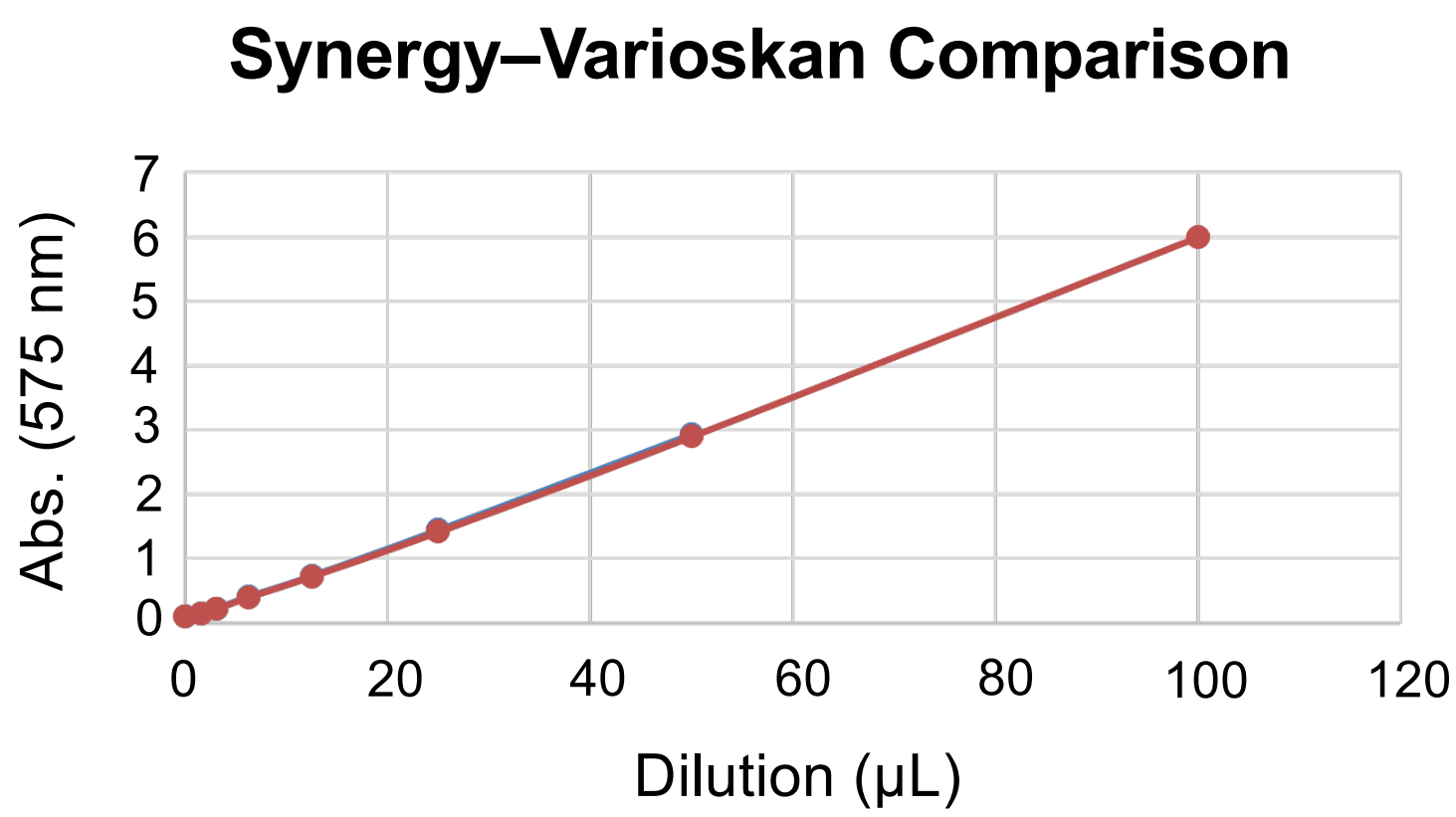

Figure S1. Plate reader validation. To evaluate assay performance across two plate readers (Synergy and Varioskan), ampicillin-induced color development was measured using serial dilutions of CPRG and ampicillin. Both instruments showed consistent absorbance profiles (Synergy, blue; Varioskan, red), confirming reproducibility and compatibility across the tested concentration ranges.

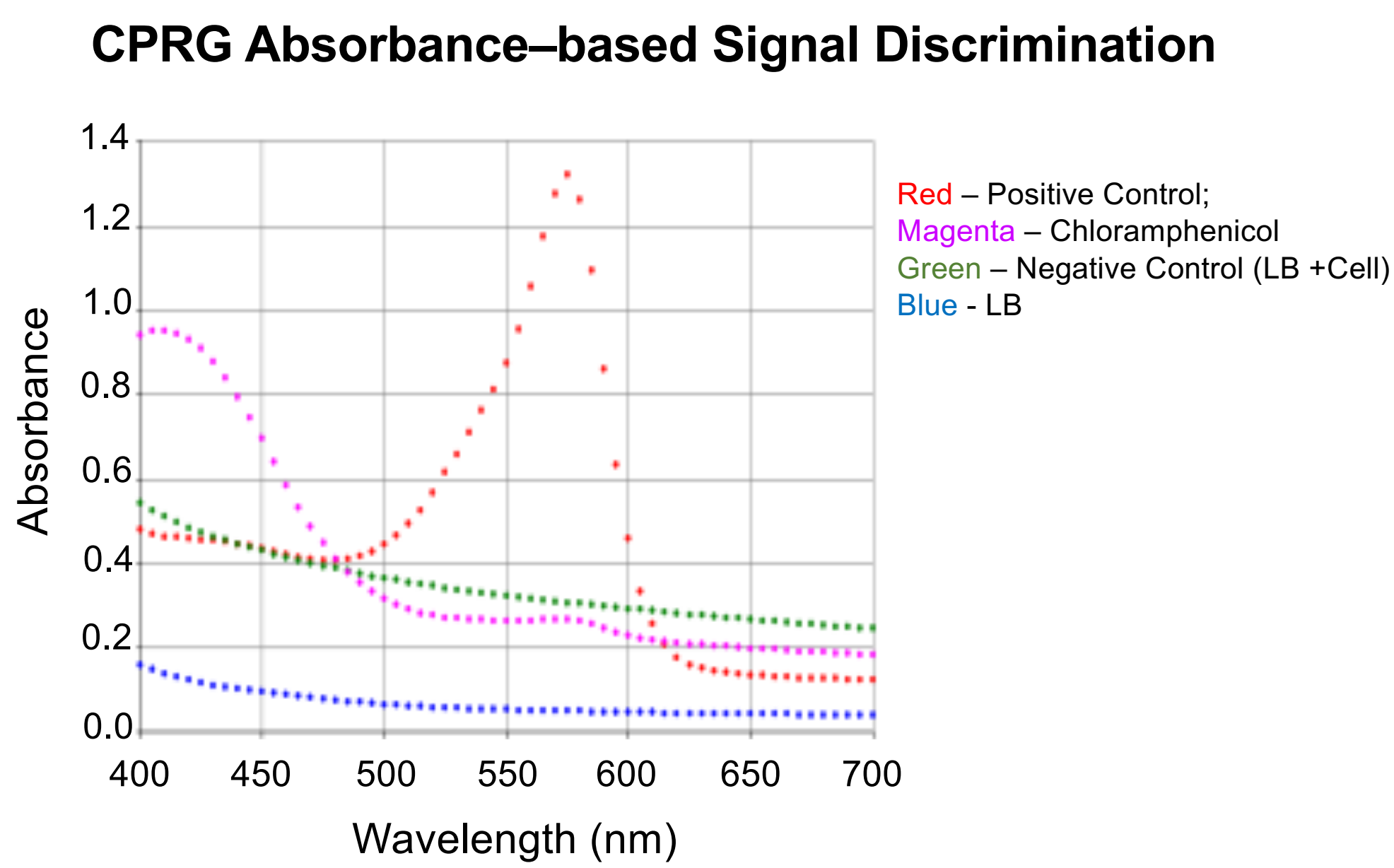

Figure S2. Signal discrimination at the CPRG absorbance readout (575 nm) using positive control (ampicillin + LB + *E. coli* + CPRG), negative/baseline control (LB + *E. coli* + CPRG), orthogonal negative control (chloramphenicol + LB + *E. coli* + CPRG), and a cell-free background blank (LB + CPRG, no bacteria).

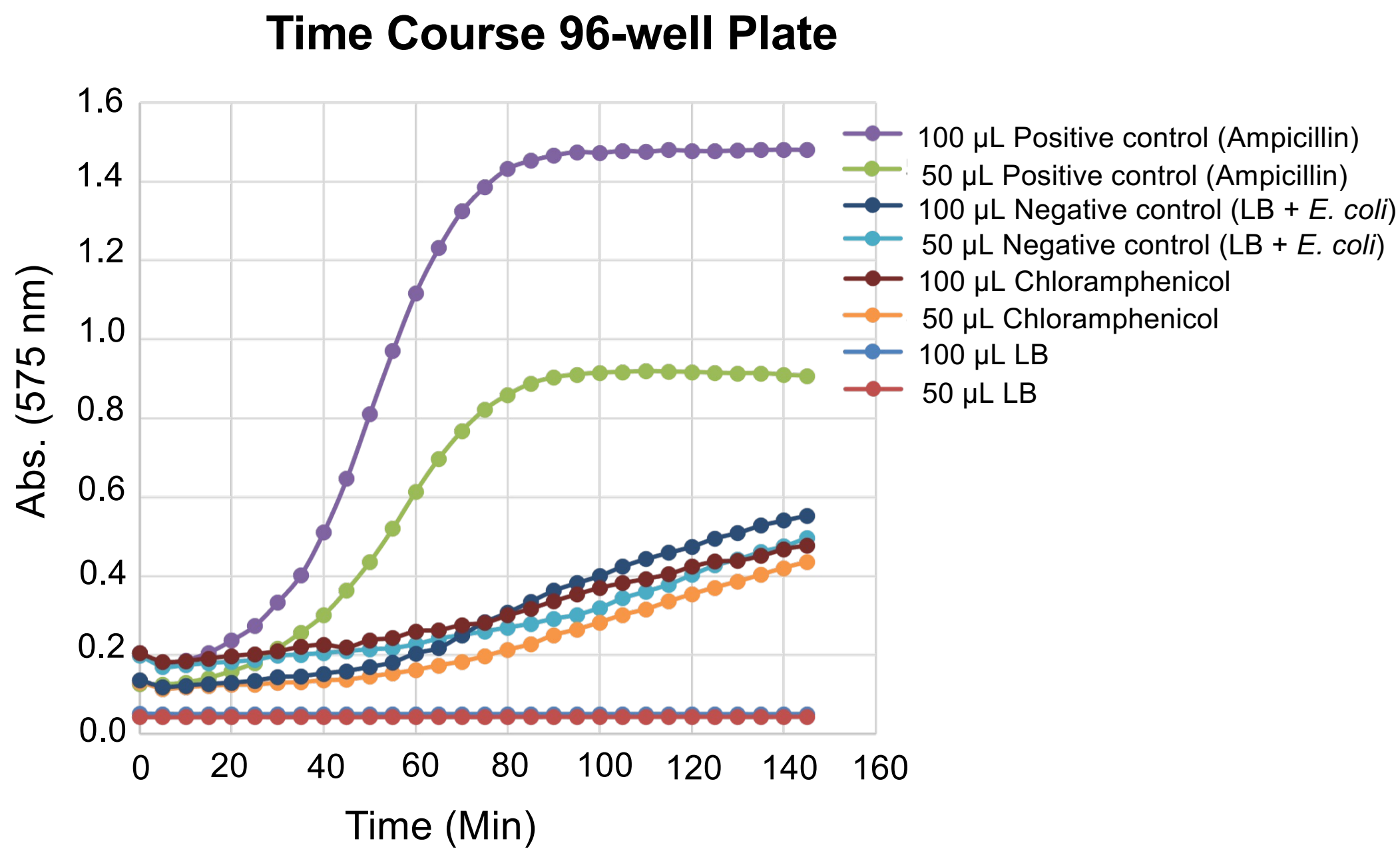

Figure S3. CPRG kinetics in the 96-well format. Absorbance at 575 nm was continuously monitored for up to 150 min using reduced reaction volumes, with additional measurements at 3 h and overnight to assess signal stability, using positive control (ampicillin + LB + *E. coli* + CPRG), negative/baseline control (LB + *E. coli* + CPRG), orthogonal negative control (chloramphenicol + LB + *E. coli* + CPRG), and a cell-free background blank (LB + CPRG, no bacteria).

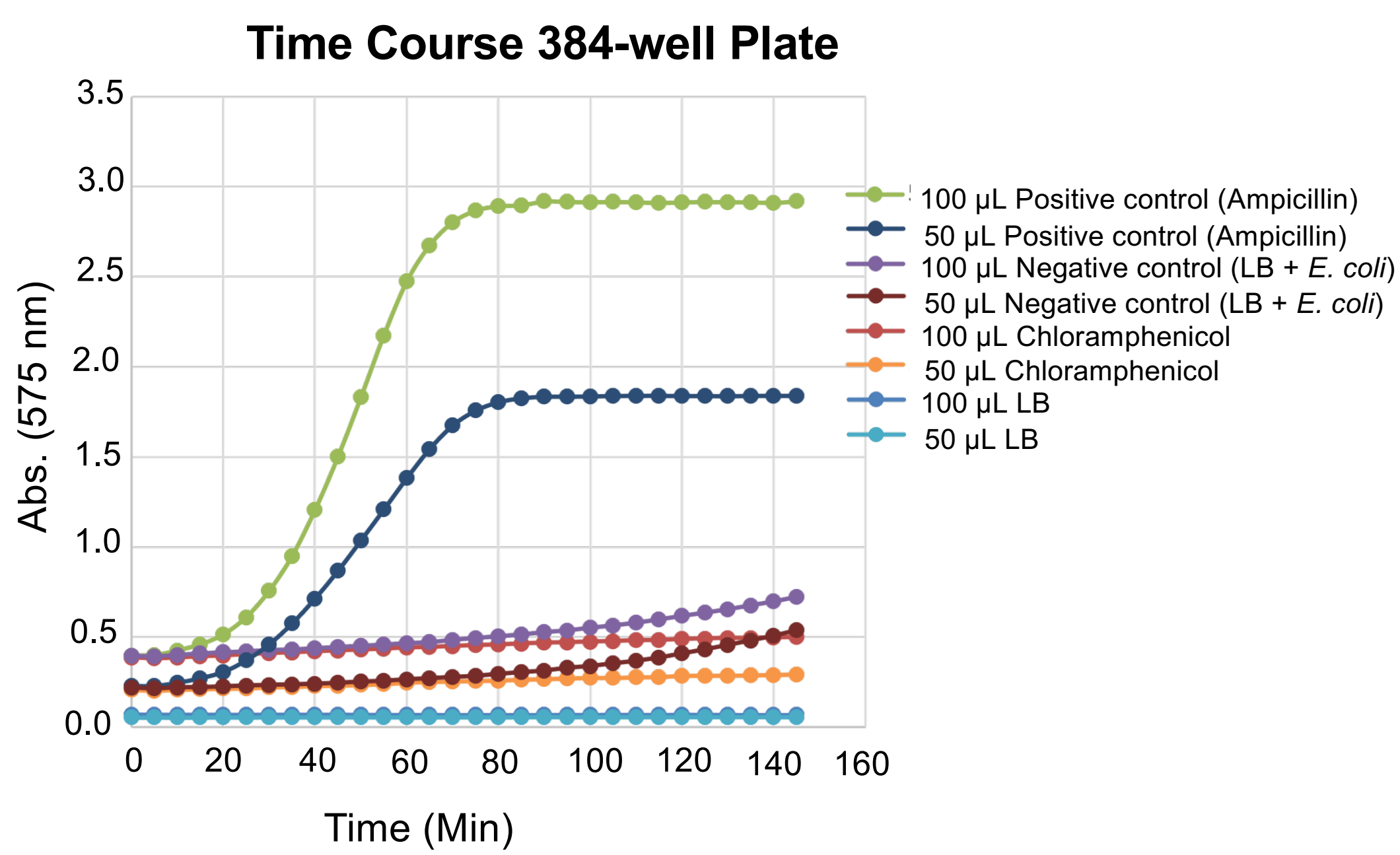

Figure S4. CPRG kinetics in 384-well format. Absorbance at 575 nm was continuously monitored for up to 150 min using miniaturized reaction volumes, with additional measurements collected at 3 h and overnight to assess signal stability. The same control set used in Figure S3 was included for assay evaluation.

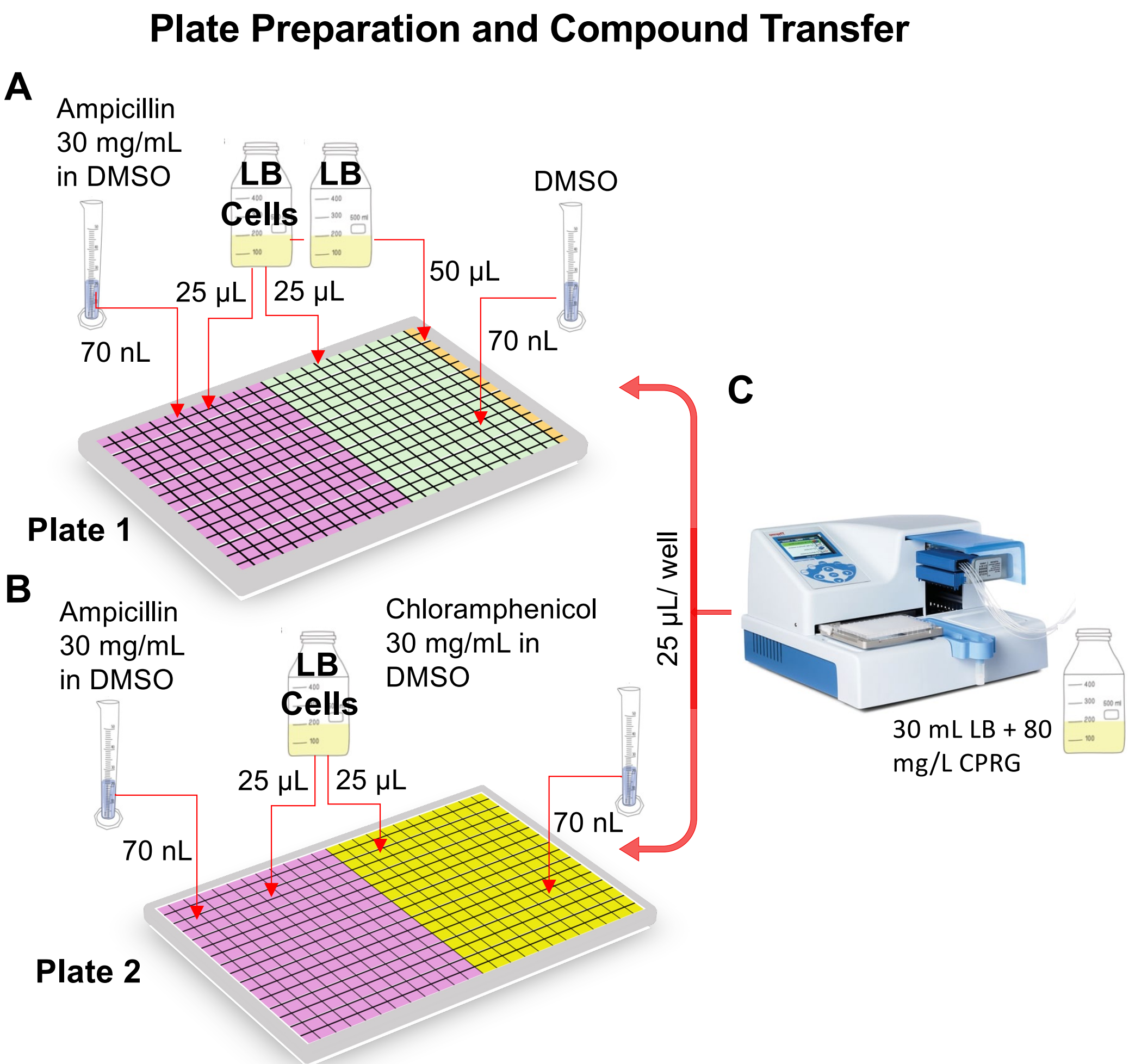

Figure S5. Plate preparation and compound transfer workflow for 384-well HTS. Two 384-well assay plates were prepared with 25 µL of LB-containing solutions per well. (A) Plate 1: one half of the plate received ampicillin (70 nL; 30 mg/mL in DMSO) as the positive control, while the other half contained untreated bacterial baseline control wells. The final column contained LB with DMSO (vehicle control). (B) Plate 2: one half of the plate received ampicillin (70 nL; 30 mg/mL in DMSO) as the positive control, and the other half received chloramphenicol (70 nL; 30 mg/mL in DMSO) as the orthogonal negative control. (C) Using a Thermo Combi liquid handler, 25 µL of LB bacterial culture supplemented with CPRG (80 mg/L) was transferred into each well of both plates, resulting in a final assay volume of 50 µL and a final CPRG concentration of 40 mg/L. Plates were incubated at room temperature with agitation, and OD575 was measured after 3 h and overnight incubation to assess rapid and delayed cell envelope permeabilization. Assay quality was evaluated using Z′-factor, signal-to-background (S/B), and signal-to-noise (S/N) values calculated from control well distributions.

96-well Absorbance Data and Plate Images

A

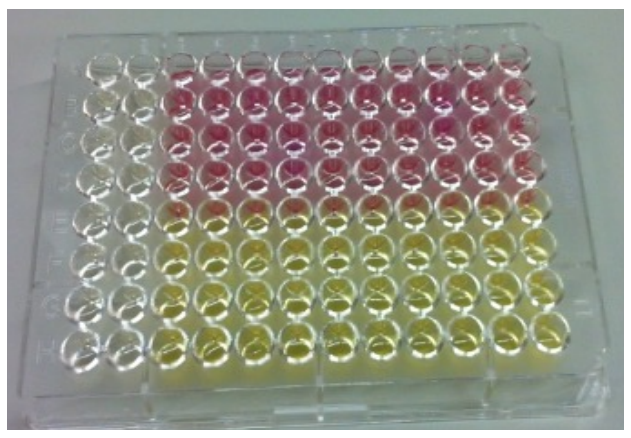

100 μL / well

Z′ = 0.6
S/B = 3.9
S/N = 41.5

B

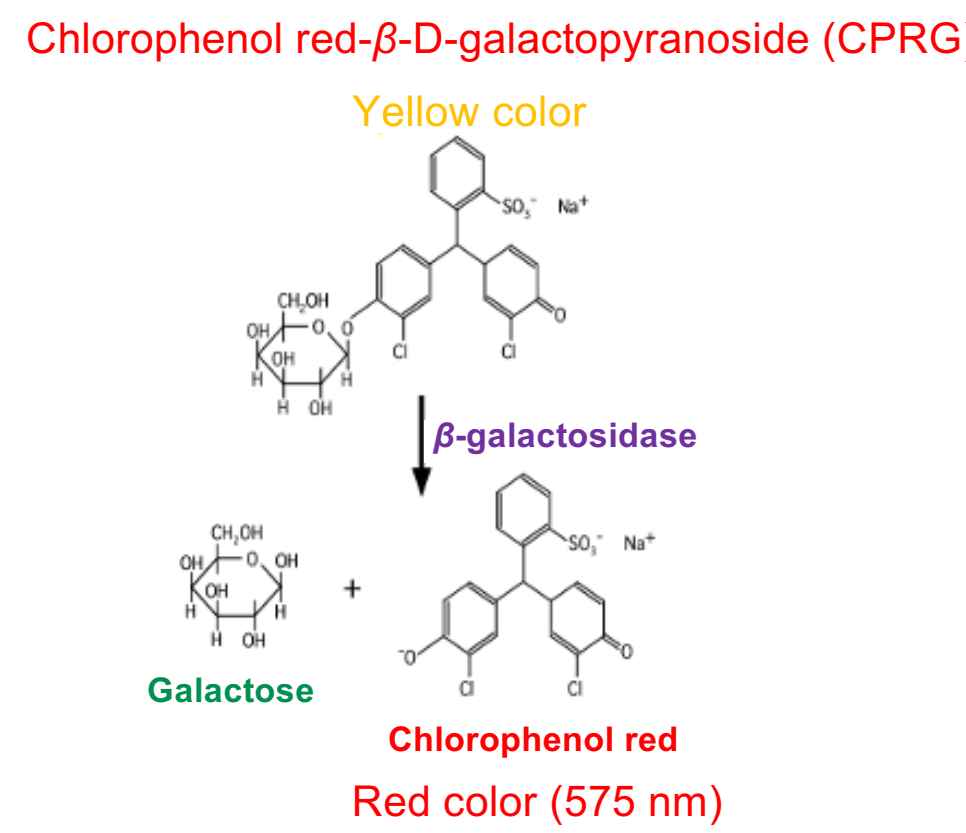

C

LB (column 1); Chloramphenicol (column 2); Ampicillin (rows A–D); LB+*E. coli* (rows E–H)

	1	2	3	4	5	6	7	8	9	10	11	12
A	0.06	0.283	1.023	1.06	1.141	1.172	1.213	1.227	1.233	1.248	1.248	1.213
B	0.048	0.307	0.88	1.096	1.178	1.139	1.157	1.173	1.188	1.351	1.255	1.275
C	0.049	0.307	0.958	1.044	1.133	1.32	1.154	1.15	1.16	1.19	1.201	1.166
D	0.051	0.29	0.84	1.005	1.067	1.075	1.099	1.185	1.165	1.175	1.193	1.187
E	0.049	0.296	0.275	0.288	0.277	0.286	0.291	0.29	0.297	0.292	0.301	0.304
F	0.049	0.267	0.271	0.286	0.324	0.302	0.29	0.288	0.286	0.293	0.305	0.303
G	0.051	0.3	0.271	0.286	0.4	0.281	0.288	0.294	0.288	0.3	0.313	0.305
H	0.049	0.307	0.266	0.266	0.283	0.272	0.308	0.275	0.281	0.29	0.3	0.324

Figure S6. 96-well absorbance data and plate images. (A) Original 96-well plate images. (B) Schematic of CPRG cleavage and colorimetric signal generation. (C) Quantitative absorbance data corresponding to the CPRG heatmaps measured at 575 nm using positive control (ampicillin + LB + *E. coli* + CPRG), negative/baseline control (LB + *E. coli* + CPRG), orthogonal negative control (chloramphenicol + LB + *E. coli* + CPRG), and a cell-free background blank (LB + CPRG, no bacteria).

384-well Absorbance Data and Plate Images

A

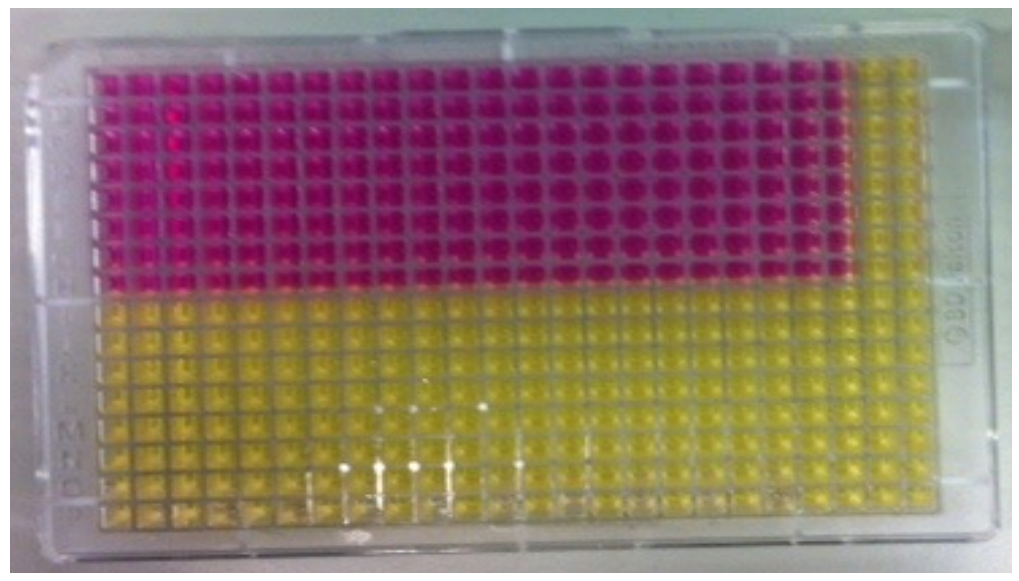

50 µL / well

Z′ = 0.9
S/B = 4.8
S/N = 194

B

Chloramphenicol (column 23); LB (column 24)

Group		1	2	3	4	5	6	7	8	9	10	11	12	13	14	15	16	17	18	19	20	21	22	23	24
Ampicillin	A	1.494	1.518	1.521	1.519	1.542	1.509	1.523	1.48	1.486	1.5	1.499	1.518	1.52	1.498	1.5	1.508	1.502	1.505	1.518	1.496	1.493	1.499	0.368	0.374
	B	1.474	1.509	1.482	1.511	1.515	1.508	1.496	1.486	1.518	1.488	1.486	1.498	1.489	1.52	1.523	1.537	1.519	1.531	1.531	1.547	1.537	1.547	0.381	0.401
	C	1.456	1.441	1.462	1.456	1.48	1.475	1.458	1.459	1.471	1.469	1.466	1.477	1.472	1.476	1.476	1.489	1.484	1.487	1.503	1.499	1.466	1.467	0.375	0.377
	D	1.459	1.449	1.456	1.464	1.476	1.477	1.466	1.476	1.48	1.476	1.477	1.478	1.476	1.483	1.485	1.486	1.49	1.481	1.503	1.502	1.499	1.502	0.377	0.389
	E	1.43	1.421	1.442	1.423	1.452	1.449	1.45	1.454	1.454	1.454	1.469	1.475	1.472	1.466	1.481	1.482	1.481	1.485	1.499	1.495	1.452	1.439	0.377	0.383
	F	1.464	1.453	1.461	1.429	1.438	1.442	1.444	1.442	1.446	1.451	1.442	1.452	1.443	1.455	1.449	1.452	1.457	1.459	1.466	1.474	1.467	1.477	0.374	0.386
	G	1.43	1.418	1.438	1.431	1.446	1.444	1.444	1.455	1.454	1.453	1.459	1.46	1.462	1.467	1.468	1.48	1.473	1.484	1.486	1.501	1.462	1.476	0.377	0.377
	H	1.465	1.447	1.463	1.459	1.431	1.44	1.441	1.439	1.443	1.446	1.439	1.446	1.449	1.453	1.453	1.444	1.456	1.454	1.465	1.467	1.461	1.478	0.374	0.382
LB+*E. coli*	I	0.3	0.302	0.309	0.306	0.308	0.306	0.307	0.315	0.308	0.301	0.31	0.305	0.313	0.305	0.316	0.315	0.316	0.312	0.32	0.306	0.319	0.311	0.376	0.377
	J	0.304	0.3	0.308	0.308	0.316	0.308	0.307	0.305	0.304	0.306	0.303	0.305	0.309	0.301	0.307	0.306	0.312	0.315	0.313	0.309	0.313	0.307	0.374	0.381
	K	0.305	0.305	0.307	0.31	0.31	0.306	0.307	0.309	0.311	0.309	0.31	0.307	0.309	0.308	0.318	0.317	0.319	0.307	0.309	0.303	0.315	0.308	0.374	0.386
	L	0.306	0.304	0.308	0.311	0.313	0.315	0.307	0.309	0.306	0.311	0.305	0.315	0.311	0.306	0.307	0.309	0.311	0.315	0.308	0.313	0.316	0.31	0.374	0.389
	M	0.303	0.301	0.308	0.305	0.308	0.306	0.308	0.315	0.305	0.307	0.308	0.302	0.31	0.308	0.311	0.31	0.31	0.301	0.305	0.304	0.315	0.305	0.37	0.382
	N	0.302	0.3	0.307	0.313	0.31	0.308	0.306	0.307	0.303	0.309	0.305	0.309	0.308	0.305	0.307	0.307	0.306	0.31	0.31	0.313	0.313	0.307	0.373	0.394
	O	0.3	0.305	0.308	0.304	0.304	0.306	0.305	0.311	0.305	0.305	0.304	0.299	0.306	0.305	0.307	0.309	0.306	0.311	0.316	0.304	0.317	0.321	0.374	0.389

Figure S7. 384-well absorbance data and plate images. (A) Original 384-well plate images. (B) Quantitative absorbance data corresponding to the CPRG heatmaps measured at 575 nm using positive control (ampicillin + LB + *E. coli* + CPRG), negative/baseline control (LB + *E. coli* + CPRG), orthogonal negative control (chloramphenicol + LB + *E. coli* + CPRG), and a cell-free background blank (LB + CPRG, no bacteria).

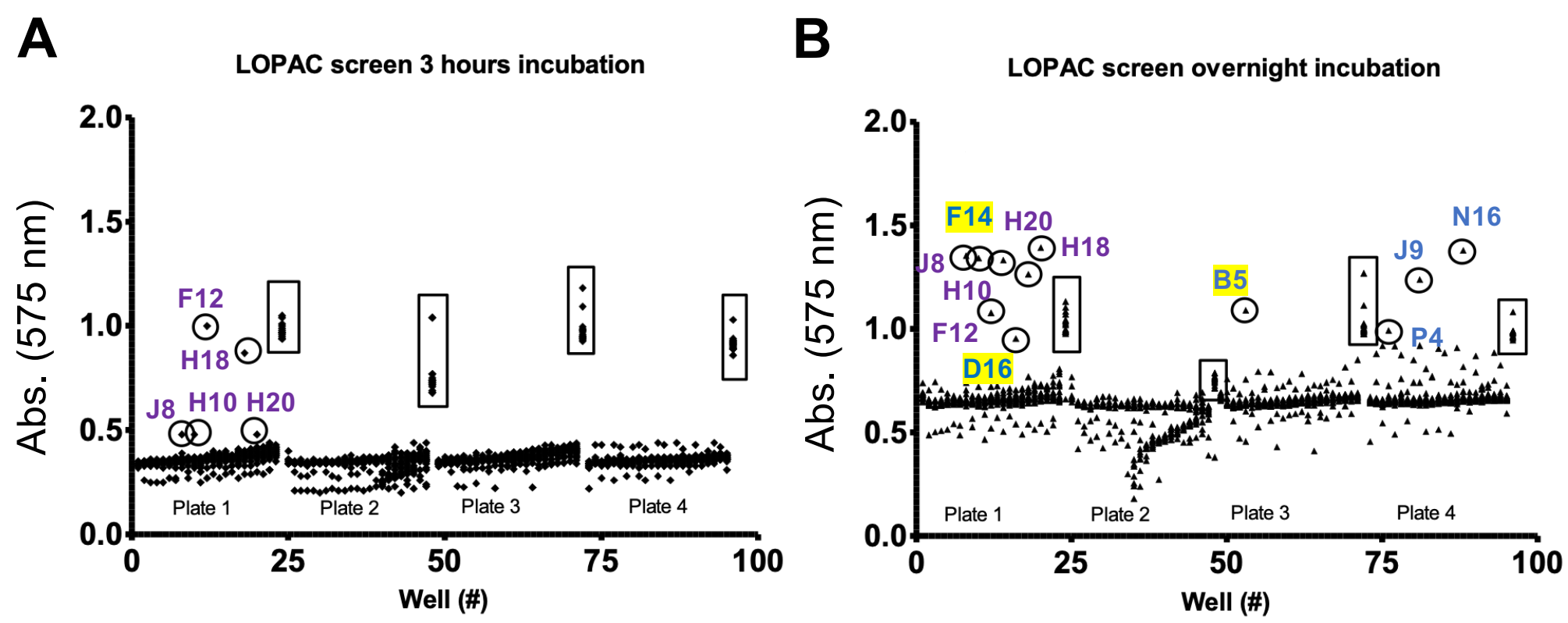

Figure S8. Distribution of LOPAC[1280] compound screening in *E. coli*. (A, B) OD_{575} measurements after 3 h (A) and overnight (B) incubation. Positive controls (ampicillin) are indicated by boxes; hit compounds are shown as circles labeled by well position (compound names in Table 2). Data were normalized to the negative/baseline control and cell-free background blank; hits were defined as a ≥25% increase in normalized CPRG signal relative to the negative control (corresponding to 3 SD above the negative-control mean), yielding five hits at 3 h (A) and eleven overnight (B). X-axis: well number (#) across 384-well plates (16 rows × 24 columns); Y-axis: OD_{575}. Each data point represents a single well measurement (one compound per well).

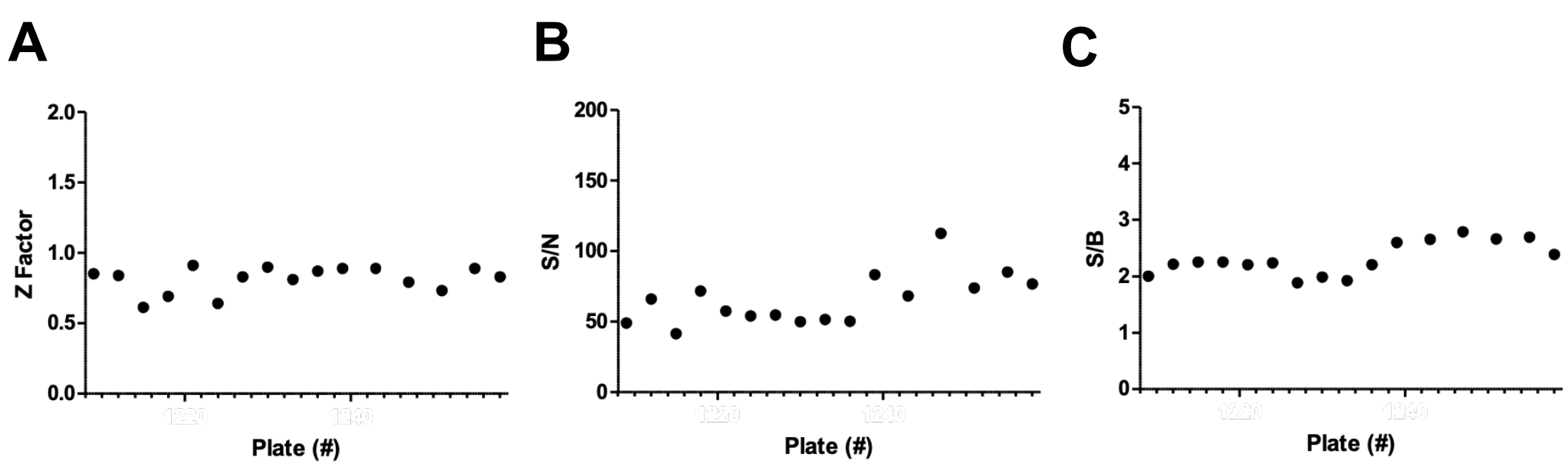

Figure S9. Quality metrics for the KD_2 high-throughput screen. Performance metrics were evaluated across the 16 assay plates (384-well format) comprising the KD_2 screen, with each point representing one plate: (A) Z′-factor, (B) signal-to-noise ratio (S/N), and (C) signal-to-background ratio (S/B). The X-axis shows plate number (1–16); the Y-axis shows the corresponding metric value. Metrics were calculated from positive and negative control wells across independent assay runs.

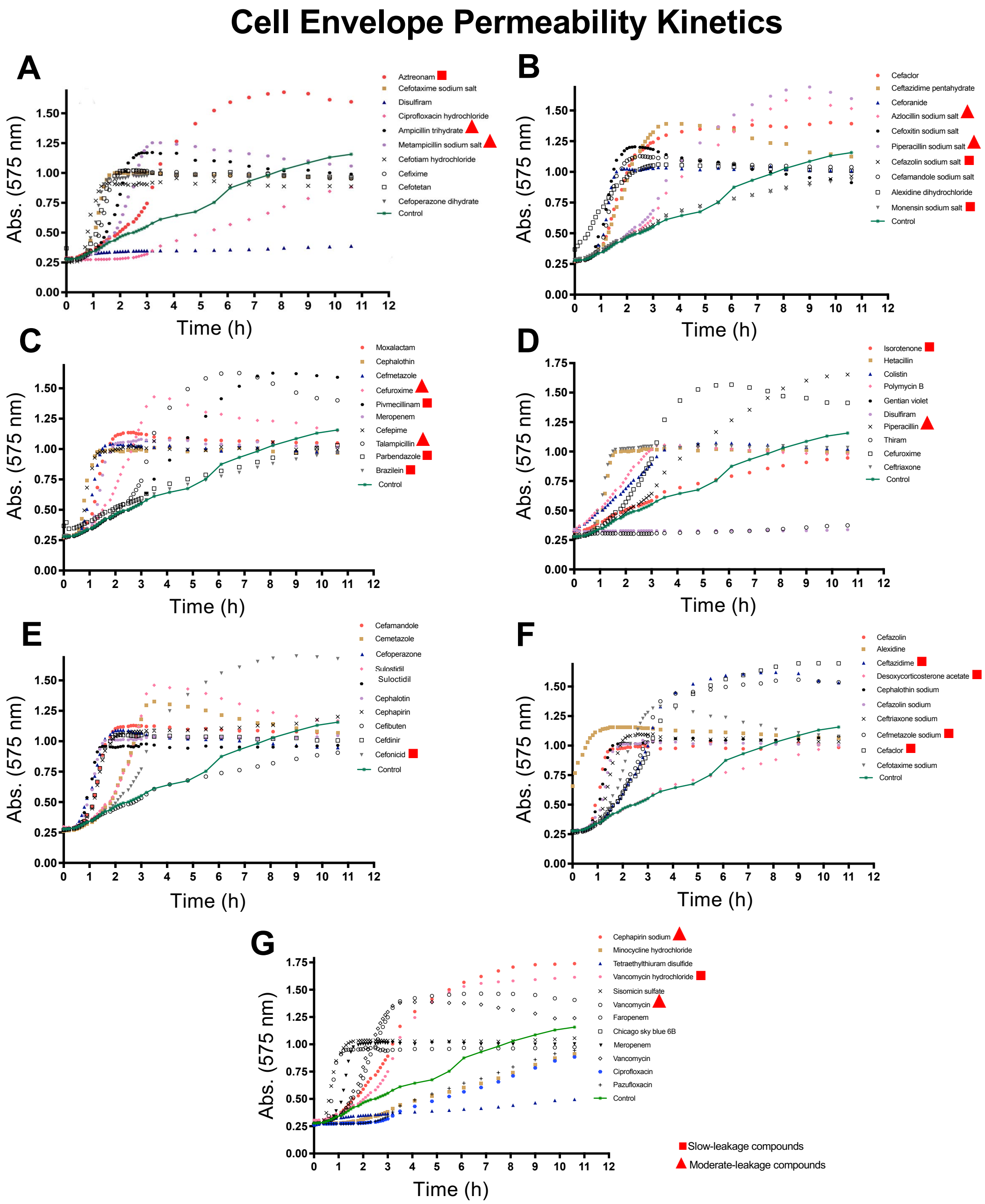

Figure S10. Progression of cell wall permeability ($A575_{nm}$) over time in the presence of compounds. LOPAC and KD activities were confirmed and organized into seven sets for clarity of display (A–G). The background signal (DMSO control) is shown as a solid green line. Absorbance at 575nm was monitored for 11 h for all active compounds. Each compound produced a distinct leakage curve. Thirteen compounds were classified as slow leakers, nine as moderate, and the remaining active compounds as rapid- acting. Leaker categories are indicated by symbols: slow (■) and moderate (▲) beside each compound; rapid compounds are unmarked. Curves represent individual compound response profiles obtained from high-throughput screening data.

Triple-staining Confocal Microscopy

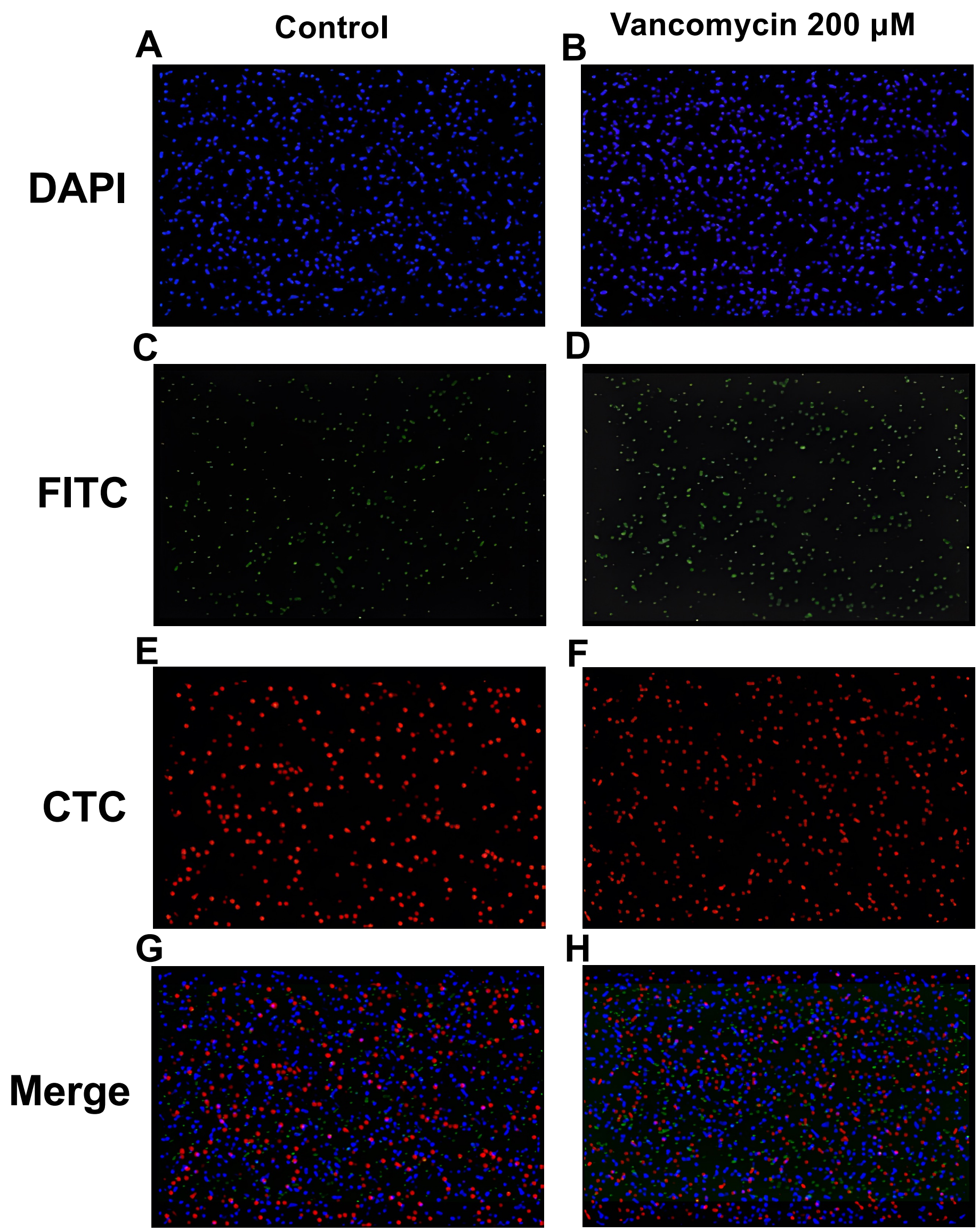

Figure S11. Validation of DAPI/FITC/CTC triple-staining confocal microscopy in *E. coli* treated with vancomycin [(0.2 mM (200 µM)]. DAPI (total cells), FITC (membrane permeabilization), and CTC (cell viability) staining of control (A, C, E) and vancomycin-treated (B, D, F) cells, with merged composite overlays shown for control (G) and vancomycin-treated (H) cells. DAPI staining (A, B) was comparable between conditions, confirming equivalent total cell counts. FITC staining appeared qualitatively increased in vancomycin-treated cells (D, H) relative to control (C, G), consistent with membrane permeabilization, while CTC staining was visibly reduced in vancomycin-treated cells (F, H) relative to control (E, G), consistent with a partial decrease in viability. Images were acquired using a 100× objective and are representative of independent experiments.

Table S1. Permeabilization constants (K_{50}) of six selected compounds.

Compound name	Permeabilization constant, K_{50} (mM) Exponential Notation	Permeabilization constant, K_{50} (μM)
Vancomycin	$(4.0 \pm 0.9) \times 10^{-2}$	40 ± 9
Cefaclor	$(7.5 \pm 1.7) \times 10^{-2}$	75 ± 17
Cefsoludin	$(7.7 \pm 3.0) \times 10^{-2}$	77 ± 30
Isorotenone	$(1.04 \pm 0.15) \times 100^{0}$	1040 ± 145
Suloctidil	$(5.87 \pm 1.00) \times 10^{-1}$	587 ± 100
Alexidine	$(2.7 \pm 0.6) \times 10^{-3}$	2.7 ± 0.6
